# Complexity of Bidirectional Transcription and Alternative Splicing at Human *RCAN3* Locus

**DOI:** 10.1371/journal.pone.0024508

**Published:** 2011-09-22

**Authors:** Federica Facchin, Lorenza Vitale, Eva Bianconi, Francesco Piva, Flavia Frabetti, Pierluigi Strippoli, Raffaella Casadei, Maria Chiara Pelleri, Allison Piovesan, Silvia Canaider

**Affiliations:** 1 Center for Research in Molecular Genetics (Fondazione CARISBO), Department of Histology, Embryology and Applied Biology, University of Bologna, Bologna, Italy; 2 Department of Biochemistry, Biology and Genetics, Polytechnic University of Marche, Monte D'Ago, Ancona, Italy; University of Florida, United States of America

## Abstract

Human *RCAN3* (regulator of calcineurin 3) belongs to the human *RCAN* gene family.

In this study we provide, with *in silico* and *in vitro* analyses, the first detailed description of the human multi-transcript *RCAN3* locus. Its analysis revealed that it is composed of a multigene system that includes at least 21 *RCAN3* alternative spliced isoforms (16 of them identified here for the first time) and a new *RCAN3* antisense gene (*RCAN3AS*). In particular, we cloned *RCAN3-1,3,4,5* (lacking exon 2), *RCAN3-1a,2,3,4,5*, *RCAN3-1a,3,4,5*, *RCAN3-1b,2,3,4,5*, *RCAN3-1c,2,3,4,5*, *RCAN3-1c,2,4,5* and *RCAN3-1c,3,4,5*, isoforms that present a different 5′ untranslated region when compared to *RCAN3*. Moreover, in order to verify the possible 5′ incompleteness of previously identified cDNA isoforms with the reference exon 1, ten more alternative isoforms were retrieved. Bioinformatic searches allowed us to identify *RCAN3AS*, which overlaps in part with exon 1a, on the opposite strand, for which four different *RCAN3AS* isoforms were cloned.

In order to analyze the different expression patterns of *RCAN3* alternative first exons and of *RCAN3AS* mRNA isoforms, RT-PCR was performed in 17 human tissues.

Finally, analyses of *RCAN3* and *RCAN3AS* genomic sequences were performed to identify possible promoter regions, to examine donor and acceptor splice sequences and to compare evolutionary conservation, in particular of alternative exon 1 or 1c - exon 2 junctions in different species.

The description of its number of transcripts, of their expression patterns and of their regulatory regions can be important to clarify the functions of *RCAN3* gene in different pathways and cellular processes.

## Introduction

*RCAN* (regulator of calcineurin) human gene family includes three members: *RCAN1* (also known as *DSCR1* - Down syndrome critical region gene 1 - [1], *MCIP1*, *CALP1*, *ADAPT78*), *RCAN2* (also known as *ZAKI-4* - [2], *DSCR1L1*, *MCIP2*, *CALP2*) and *RCAN3* (also known as *DSCR1L2* - [3], *MCIP3*, *CALP3*), the studied gene in the present work.

The novel denomination for *RCAN* genes and proteins has recently been approved by the HUGO Gene Nomenclature Committee, and due to the large number of human *RCAN* mRNA isoforms, a specific nomenclature was proposed: *RCAN1*, *RCAN2* or *RCAN3* - followed by the hitherto identified exon numbers [4].

All three *RCAN* gene products have been demonstrated to interact with and inhibit calcineurin [5]–[7], a Ca^2+^/calmodulin-activated serine/threonine phosphatase that is involved in the transcriptional activation of many target genes. In particular, calcineurin activation causes nuclear factor of activated T-cells (NFAT) transcription factors translocation to the nucleus, where, in cooperation with other transcription factors, they induce genes expression. A calcineurin inhibitor RCAN motif (RCAN CIC) has been demonstrated to bind calcineurin [8], whose signalling plays a role in many physiological and pathological processes, including cardiac hypertrophy [9], [10], T-cell activation and cytokine gene expression [11], [12], skeletal myocyte differentiation and fibre-type switching [13],[14], synaptic plasticity or neurotransmission [15], [16], cell apoptosis [17] and endometrial adenocarcinoma regulation [18]. Recently, the *RCAN3* inhibitory role on human umbilical vein endothelial cells (HUVEC) proliferation, both basally and under vascular endothelial growth factor or phorbol 12-myristate 13-acetate stimulation conditions, has been demonstrated. This process is probably mediated by calcineurin signalling and independent from the inflammatory and angiogenic processes [19]. Moreover, we demonstrated that RCAN3 also interacts with TNNI3 [20], the human inhibitory cardiac troponin that prevents contraction in the absence of calcium and troponin C [21]. *RCAN3* exon 2 product has been found to be sufficient for binding TNNI3 [20], [22].

*RCAN3* is the most recently identified member of the human *RCAN* gene family, appearing only in vertebrates, in agreement with the fact that the number of *RCAN* members tends to increase in more complex organisms [23]. *RCAN3* gene (GenBank accession number: NM_013441) [24] is localized on chromosome 1 (1p36.11), it is composed of five exons and the coding sequence, included between exon 2 and exon 5, encodes for a 241 amino acid predicted protein (estimated molecular weight 27.5 kilodalton - kDa), which shares a highly conserved consensus motif, known as the FLISPP motif, comprising the signature of the family [3]. Due to the fact that *RCAN3* mRNA is the first identified isoform, in the present work we refer to *RCAN3* as the reference isoform under the name *RCAN3-1,2,3,4,5*, to distinguish it from isoforms with a different first exon. To date, four alternative splicing isoforms have been identified: *RCAN3-2,3,4b,5* (GenBank accession number: AF176117), which presents an in-frame loss of 30 bases with consequent absence of 10 amino acids in the middle of the predicted protein [3]; *RCAN3-2,5*, lacking exons 3 and 4 (GenBank accession number: AY906854) [20]; *RCAN3-2,4,5* (GenBank accession numbers: EF431960 and EF529733), lacking exon 3; and *RCAN3-2,3,5* (GenBank accession number: EF467309), lacking exon 4 [22]. All identified isoforms were expressed in the 7 investigated human tissues (heart, brain, small intestine, lung, testis, prostate, and peripheral blood leukocytes), with a constant expression prevalence of *RCAN3-1,2,3,4,5* [22]. All alternative protein isoforms interact with TNNI3, as demonstrated by a glutathione-sepharose transferase fusion protein assay by using the exon 2 encoded domain [20], [22], while only RCAN3-1,2,3,4,5, RCAN3-2,3,4b,5 and RCAN3-2,4,5 show the CIC motif, necessary for calcineurin binding. In fact, *RCAN3-2,5* and *RCAN3-2,3,5* show a frameshift in the exon 5-encoded amino acid sequence, thus encoding for isoforms with different C-terminal regions that lack the CIC motif, whose first five amino acids are encoded by exon 4 and the remaining by exon 5 [22].

Since preliminary bioinformatic data revealed a not yet investigated major complexity of the human *RCAN3* locus, an accurate multiple approach analysis of the locus was considered necessary. Therefore, the aim of the present work is a combination of *in silico* and *in vitro* analyses in order to explore as much as possible the complexity of the human *RCAN3* locus, focusing on several new human *RCAN3* spliced isoforms, on a new gene overlapped and in antisense compared to *RCAN3*, as well as on new *RCAN3* isoforms with different 5′ untranslated regions (UTRs). Our combined data from this study indicate the presence of a complex multi-transcript system at the *RCAN3* locus.

Analysis of this locus and reverse transcription polymerase chain reaction (RT-PCR) cloning experiments led us to identify 16 new *RCAN3* putative spliced isoforms, to be added to the five already known. The new isoforms differ in both coding exons and non-coding 5′UTR first exons. They were: *RCAN3-1,3,4,5*, *RCAN3-1a,2,3,4,5*, *RCAN3-1a,3,4,5, RCAN3-1b,2,3,4,5*, *RCAN3-1c,2,3,4,5*, *RCAN3-1c,3,4,5*, *RCAN3-1,2,4*, *RCAN3-1a,2,4*, *RCAN3-1b,2,4*, *RCAN3-1c,2,4,5*, *RCAN3-1,2,3,5*, *RCAN3-1a,2,3,5*, *RCAN3-1c,2,3,5*, *RCAN3-1,2,5, RCAN3-1a,2,5* and *RCAN3-1c,2,5*. Bioinformatic analysis allowed us also to identify the human *RCAN3AS* gene, the *RCAN3* antisense gene, which in part overlaps with *RCAN3-1a,2,3,4,5* on the opposite strand. The 4 *RCAN3AS* isoforms (*RCAN3AS-1,2,3*, *RCAN3AS-1,2a,3*, *RCAN3AS-1,2b,3* and *RCAN3AS-1,3*) denomination was assigned according to the HUGO Gene Nomenclature Committee. Moreover, RT-PCR was performed in order to analyze the expression patterns of *RCAN3* alternative non-coding first exons (1, 1c, 1a and 1b) and *RCAN3AS-1,2,3* mRNA isoforms in 17 different human tissue types.

Furthermore, *RCAN3* and *RCAN3AS* mRNA isoforms and amino acid motif analyses were performed by mRNA and protein multiple alignments, respectively. Since the evidence of alternative 5′UTR first exons, analysis of *RCAN3* and *RCAN3AS* genomic sequences were conducted to predict possible different promoter regions. Finally, genomic sequences were used to study alternative donor and acceptor splice sequences and to compare the evolutionary conservation of alternative non-coding first exon 1 or 1c - exon 2 junctions in different species.

## Materials and Methods

### Reverse transcription - Polymerase chain reaction (RT-PCR)

Standard reverse transcription conditions were: 2 g total RNA, Moloney murine leukaemia virus reverse-transcriptase (Promega, Madison, WI; used with companion buffer) 400 U, oligo dT-15 2.5 µM, random hexamers 2 µM, dNTPs 500 µM each. RT reaction was performed in a final volume of 50 µL for 60 min at 37°C. Standard PCR conditions for all amplifications were: 25 µL final volume, primers 0.3 µM each, 12.5 µL BioMix Red (Bioline, Taunton, MA); initial denaturation of 2 min at 94°C; 45 cycles of 30 s at 94°C, 30 s at the indicated annealing temperature (T_a_), 30 s at 72°C; final extension of 7 min at 72°C. Deviations from these conditions are given in details when appropriate.

### RT-PCR cloning and plasmid construction

RT with standard conditions was used to obtain DNA complementary to RNA (cDNA) from commercial human total RNAs (Clontech, Palo Alto, CA). All used primers were designed using Amplify software [25], following standard criteria [26].

Specific primers were used to clone *RCAN3-1,2,3,4,5* (Table 1, #1 and #2) cDNA from 5 µL of human peripheral blood leukocytes cDNA using PCR standard conditions with a T_a_ of 61°C. Specific primers (Table 1, #3 and #4 or #2 as reverse primers) were used to clone *RCAN3-1a,2,3,4,5* cDNA from 3 µL of human prostate cDNA using PCR standard conditions, except that there were 30 µL of final volume and 40 cycles with a T_a_ of 63°C. Specific primers (Table 1, #5 and #6 or #2 as reverse primers) were used to clone *RCAN3-1b,2,3,4,5* cDNA from 5 µL of human lung and testis cDNAs using PCR standard conditions with a T_a_ of 63°C. Primer #2, projecting on exon 5 of *RCAN3* locus, was used for *RCAN3-1a,2,3,4,5* and *RCAN3-1b,2,3,4,5* cloning, to account for the possible incompleteness in 3′ of the relative expressed sequence tag (EST) reference sequences. Specific primers (Table 1, #7 and #8) were used to clone *RCAN3AS* cDNAs from 3 µL of human testis and pancreas cDNAs using PCR standard conditions, except that there were 30 µL of final volume and 35 cycles with a T_a_ of 61°C. To demonstrate the possible presence of exon 1 in all identified *RCAN3* isoforms to date, 5 µL of human peripheral blood leukocytes cDNA was used in a standard PCR with a T_a_ of 61°C. The data source for primers design were the four GenBank RNA sequences previously described by Facchin et al. 2008 [22] (*RCAN3-2,3,4b,5* - GenBank accession number: AF176117, *RCAN3-2,5* - accession number: AY906854, *RCAN3-2,4,5* - accession numbers: EF431960 and EF529733, and *RCAN3-2,3,5* - accession number: EF467309) and the *RCAN3* reference sequence containing the non-coding exon 1 (GenBank accession number: NM_013441). To clone the *RCAN3* alternative isoforms, a forward primer common to all gene isoforms on exon 1 (Table 1, #1) and reverse primers specific for *RCAN3-2,3,4b,5*, *RCAN3-2,5*, *RCAN3-2,4,5* and *RCAN3-2,3,5* isoforms (Table 1, #9, #10, #11, #12, respectively) were designed. Each of these reverse primers presented two mismatches in the last three bases of the 3′ end (one being the 3′-residue) compared to the sequence of the other isoforms. Reverse primers were designed encompassing specific exon - exon boundaries (Table 1).

**Table 1:** Primer list.

No	Primer sequence 5′ → 3′	gene or plasmid c	specificity d
#1	CTCCCGTCCTGCGGCGGCGGGGCGT (F)^a^	*RCAN3-1*	exon 1/exon 1c
#2	CAGAGTCTCACCTATGCTGTTCG (R)^b^	*RCAN3* isoforms	exon 5
#3	GTGCTAGCTCCAGCTCAGCTACCT (F)	*RCAN3-1a*	exon 1a
#4	AGATAGGACTTGTCCCGCACTTCGC (R) (R)	*RCAN3-1a* *	exon 4
#5	GTATAACAGAAAAATGAACCAAGCACT (F)	*RCAN3-1b*	exon 1b
#6	CCATCATCTCATCCAAATCATCTTC (R)	*RCAN3-1b* *	exon 2
#7	GCGGCCGCAGGTAGCTGAGCTG (F)	*RCAN3AS*	exon 1
#8	ATGCTTTGTAGAAGTACTACAGGGA (R)	*RCAN3AS*	exon 3
#9	TGGGGCGGCAGGAGATAGGACTGTG (R)	*RCAN3-2,3,4b,5*	exon 4b-exon 3
#10	TCCCGCGTGAAGTTCATATTTCTCTCCTTC (R)	*RCAN3-2,5*	exon 5-exon 2
#11	CACTTCGCCGGACATCTGCACCTTC (R)	*RCAN3-2,4,5*	exon 4-exon 2
#12	CGTGAAGTTCATATTTCTCTCCTGTGCAA (R)	*RCAN3-2,3,5*	exon 5-exon 3
#13	AGGGTTTTCCCAGTCACGAC (F)	*pcR 2.1* plasmid	
#14	CAGGAAACAGCTATGACCATGA (R)	*pcR 2.1* plasmid	
#15	CGCCCGGCCTCTCCAGCAGGAAAG (F)	*RCAN3-1,3,4,5*	exon 1-exon 3
#16	CGGCGGCGGGGCGTGCAGGAAAG (F)	*RCAN3-1c,3,4,5*	exon 1c-exon 3
#17	TTGCCGGGAGGTGAACCGAGGAAAG (F)	*RCAN3-1a,3,4,5*	exon 1a-exon 3
#18	GATTTCTGGCACGATAAATGAGAAAG (F)	*RCAN3-1b,3,4,5*	exon 1b-exon 3
#19	GCTGATGGCGATGAATGAACACTG (F)	5′ Race Outer	
#20	TCACAGACATGAACCACCACGCTG (R)	*RCAN3* isoforms	exon 5
#21	CGCGGATCCGAACACTGCGTTTGCTGGCTTTGATG (F)	5′ Race Inner	
#22	TGTCGACTCTGTTCCCGCGTGAAG (R)	*RCAN3* isoforms	exon 5
#23	CTTCGCCGGACATCTGCACCTGTGCAA (R)	*RCAN3-1b* *	exon 4-exon 3
#24	TGCGGCGGCGGGGCGTGCAGGTGAG (F)	*RCAN3-1*	exon 1
#25	GTCCTGCGGCGGCGGGGCGTGCAGTGG (F)	*RCAN3-1c*	exon 1c-exon 2
#26	GCTACACAGATCTGACTGGCTATCATTC (R)	*RCAN3-1/1c* *	exon 2

a F, forward primer.

b R, reverse primer.

c *RCAN3-1*: isoforms with exon 1; *RCAN3-1a*: isoforms with exon 1a; *RCAN3-1b*: isoforms with exon 1b; *RCAN3AS*: all *RCAN3AS* isoforms;

*: star indicates reverse primers used for specific alternative first exon isoforms cloning, but useful to clone all isoforms containing exon 2 or exon 3 or exon 4.

d When two exon numbers are provided, the primer encompasses the boundary between the two exons.

Moreover, in order to verify the possible presence of also exon 1a or exon 1b in all identified *RCAN3* isoforms previously listed, 2 µL of human prostate and lung (for *RCAN3-1a* and *RCAN3-1b* isoforms, respectively) cDNAs were used in PCR standard conditions, except that 40 cycles with a T_a_ of 65°C were performed, with exon 1a or 1b specific forward primers (Table 1, #3 and #5, respectively) and *RCAN3* isoform specific reverse primers (Table 1, #9, #10, #11, #12).

RT-PCR products were gel-analyzed following standard methods [27]. PCR products that revealed more than one band on gel were first cloned in pCR2.1 plasmid by TA Cloning Kit (Invitrogen, Carlsbad, CA, USA) and the obtained plasmids were transformed in chemically competent TOP10 *E. coli* cells (Invitrogen). To check the plasmid inserts sequences, PCR products were obtained from 5 µL of a bacterial colony resuspended in 50 µL of water under standard PCR conditions (see subsection RT-PCR), except that there were 25 cycles with a T_a_ of 58°C and using vector-specific forward and reverse primers (Table 1, #13 and #14, respectively). These products were then sequenced as described in below subsection, with the same primers used for PCR.

To verify the existence of the *RCAN3* isoforms lacking exon 2 (*RCAN3-1,3,4,5, RCAN3-1c,3,4,5, RCAN3-1a,3,4,5* and *RCAN3-1b,3,4,5*), four specific forward primers encompassing the boundary between the alternative first exon (exons 1, 1c, 1a, 1b) and exon 3 were designed (Table 1, #15, #16, #17 and #18, respectively). In order to be isoform-specific, only five bases were designed on the exon 3, while the most part of the primer (at least 18 bases) was complementary to the first alternative exon. These forward primers were used with a common reverse primer designed on exon 5 (Table 1, #2). To clone these *RCAN3* isoforms cDNAs, PCR experiments were performed from 2.5 µL of peripheral blood leukocytes cDNA (to clone *RCAN3-1,3,4,5* and *RCAN3-1c,3,4,5*), 2.5 µL of prostate cDNA (to clone *RCAN3-1a,3,4,5*) and 5 µL of lung cDNA (to clone *RCAN3-1b,3,4,5*) using PCR standard conditions, except that 40 cycles with a T_a_ of 63°C were performed in a final volume of 25 µL.

### 5′ RLM-RACE

5′-RLM-RACE (Rapid Amplification of 5′ cDNA end) was performed using the FirstChoice RLM-RACE kit (Ambion, Austin, TX) following the manufacturer's instructions. Starting from human prostate RNA (Clontech), cDNA to use in 5′ RACE reactions was obtained. 1 µL of prostate cDNA was used in first-round 5′ RACE reaction using the 5′ Race Outer primer (#19) and target gene-specific primer designed on *RCAN3* exon 5 (#20). PCR conditions for amplification were: 25 µL final volume, primers 0.3 µM each, 12.5 µL BioMix Red (Bioline, Taunton, MA); initial denaturation of 2 min at 94°C; 25 cycles of 30 s at 94°C, 30 s at 65°C, 120°s at 72°C; final extension of 7 min at 72°C. The second-round 5′ RACE reaction used 1 µL of the first-round reaction and internal primers (5′ Race Inner primer #21 and gene-specific nested #22) with cycling as above. 10 µL of PCR products were gel-analyzed following standard methods [27], were cloned using the TA Cloning kit and sequenced as described in the following subsection.

### DNA sequencing and sequence analysis

All sequences were determined using the Big Dye Terminator Cycle Sequencing-Ready Reaction kit and automated DNA sequence analyzer ABI-PRISM 3730 (Applied Biosystems, Foster City, CA, USA). Sequences were analyzed by BLAST (Basic Local Alignment Search Tool) family programs - accessed via the NCBI (National Center for Biotechnology Information) homepage (http://www.ncbi.nlm.nih.gov/) [28] - with default parameters, using the following GenBank divisions: “nr” (non redundant) and “human ESTs” database sequences, in September 2010. Each new isoform was sequenced twice using two independent amplification reaction products as template.

### RT-PCR expression analysis

Commercial total RNAs (Clontech) obtained from 17 adult human whole normal tissues were used to clone *RCAN3-1a,2,3,4,5, RCAN3-1b,2,3,4,5* and *RCAN3AS* isoform cDNAs and to analyze their different tissue expression profile: brain (pooled from 2 male Caucasians), prostate (pooled from 16 male Caucasians), peripheral blood leukocytes (pooled from 13 male/female Caucasians), thymus (pooled from 2 male Caucasians), spleen (pooled from 13 male/female Caucasians), stomach (pooled from 50 male Caucasians), colon (pooled from 23 female Caucasians), bone marrow (pooled from 3 male Asians), testis (pooled from 45 Caucasians), lung (pooled from 3 male/female Caucasians), small intestine (pooled from 2 male/female Caucasians), adrenal gland (pooled from 62 male/female Caucasians), mammary gland (pooled from 27 female Caucasians), liver (from a 51-year old male Caucasian), pancreas (pooled from 35 male Caucasians), heart (pooled from 10 male/female Caucasians) and skeletal muscle (pooled from 7 male/female Caucasians). To clone *RCAN3-1a,2,3,4,5, RCAN3-1b,2,3,4,5* and *RCAN3AS* isoforms primers #3 and #4, #5 and #23, #7 and #8 (Table 1) were used, respectively, and PCR reactions were performed as described in the “RT-PCR cloning and plasmid construction” subsection, except for *RCAN3-1a,2,3,4,5* PCR that was performed with 32 cycles. Reverse primer #23 was designed overlapping specific exon 4 - exon 3 boundaries. The same tissues were used to clone exon 1 - exon 2 and exon 1c - exon 2 junction cDNAs from 3 µL of human cDNAs using the forward primers #24 and #25 (Table 1), respectively, and a common reverse primer designed on exon 2 (#26, Table 1). Primer #24 was designed to be specific for the exon 1, while primer #25 was designed encompassing specific exon 1c - exon 2 boundaries. PCR was performed in standard conditions, except that there were 35 cycles with a T_a_ of 63°C. All five PCRs were qualitative, but they were performed at the maximum distance from the PCR reaction plateau, that is at the lowest cycle number where the product was shown. Tissues choice was driven by the need to investigate the largest panel of tissues and by the availability of human commercial RNA in our laboratory.

### Bioinformatic analysis of mRNA and protein isoforms

The nr/nt, refseq_rna and EST databases at NCBI were searched by BLASTN software (default parameters, with no filter) for the presence of any sequence relating to the alternative *RCAN3* and *RCAN3AS* mRNA isoforms. The search was performed in all organisms. In order to do this, for all human *RCAN3* isoforms an alternative first exon - exon 2 or alternative first exon - exon 3 query sequence was built to demonstrate the different use of the alternative first exons: 1 or 1c or 1a or 1b. A following manual analysis of the found sequences allowed us to attribute them with high probability to a specific isoform. Then refseq_genomic and other genomic sequence databases at NCBI [“NCBI genomes”, “High throughput genomic sequences (HTGS)”, “Genomic survey sequences (gss)” and “Whole-genome shotgun reads (wgs)”] were searched by BLASTN (default parameters, with no filter, excluding *Homo sapiens*) for the presence of any sequence relating to human alternative first exons - exon 2 or alternative first exons - exon 3 junctions. ECgene Browser (http://genome.ewha.ac.kr/ECgene/) [29] and Genome Browser at UCSC (http://genome.ucsc.edu/cgi-bin/hgGateway) [30] were searched for the presence of any sequence relating to the studied human *RCAN3* and *RCAN3AS* mRNA isoforms. The analysis was carried out in September 2010. Multiple alignment analysis of RCAN3 protein isoforms, *RCAN3* and *RCAN3AS* isoform mRNA sequences were performed using ClustalW 1.83 software (http://www.ebi.ac.uk/clustalw) [31]. BLASTP software (default parameters, with no filter) was used to search for domain similarity.

The genomic organization of human *RCAN3* isoforms with alternative first exons and of human *RCAN3AS* isoforms were studied to compare splice donor and acceptor sequences with the consensus splice reference sequences [32]. The analysis was carried out for different *RCAN3* isoforms aligning alternative exons 1 - first intron - exon 2 or 3 (according to the considered isoform). The same study was performed comparing first exon - first intron - alternative exons 2 or 3 for *RCAN3AS* gene isoforms.

Finally, search of putative promoter regions by analysis of the genomic sequences upstream 5′ ends of the alternative first exons of *RCAN3* and *RCAN3AS* was performed by using the Neural Network Promoter Prediction version 2.2 of Berkeley Drosophila Genome Project (http://www.fruitfly.org/seq_tools/Promoter.html) [33], the Neural Network Promoter Prediction Server of BIOSINO (Bioinformation Center of Shanghai Institutes for Biological Science Chinese Academy of Sciences, http://Promoter.biosino.org) [34] and the First Exon Finder software (http://rulai.cshl.org/tools/FirstEF/) [35]. Manual adjustment was added to carry out promoter analysis.

### Comparative sequence analysis between *RCAN3-1,2,3,4,5* and *RCAN3-1c,2,3,4,5* in all organisms

The nr/nt databases at NCBI were searched with BLASTN and/or TBLASTN software (default parameters, with no filter, excluding *Homo sapiens*) for the presence of any sequence relating to human *RCAN3-1,2,3,4,5* mRNA (NM_013441) and relative protein (NP_038469), which were used as initial query sequences to retrieve other organism transcripts.

For different species, more complete retrieved transcript sequences were used as reference to query refseq_genomic and other genomic sequence databases at NCBI [“NCBI genomes”, “High throughput genomic sequences (HTGS)”, “Genomic survey sequences (gss)” and “Whole-genome shotgun reads (wgs)”] by BLASTN software (default parameters, with no filter, excluding *Homo sapiens*). In order to analyze the conservation of exon 1 - exon 2 and exon 1c - exon 2 sequences, the exon 1 - first intron - exon 2 and/or exon 1c - first intron - exon 2 sequences were annotated on the species-specific reference genomic sequences. In some cases, when the retrieved reference transcript sequences were incomplete and did not match exon 1 or 1c, the attribution of the identified transcripts to the *RCAN3* isoform containing alternative exon 1 or exon 1c was made, after a comparison between genomic sequence of studied species and reference transcript sequence of an evolutionarily related species. This allowed us to manually assemble putative species-specific exon 1 - exon 2 or exon 1c - exon 2 sequences (*Homo sapiens* transcript used for *Macaca mulatta*, *Macaca mulatta* transcript used for *Callithrix jacchus*, *Canis lupus familiaris* transcript used for *Ailuropoda melanoleuca* and *Mus musculus* transcript used for *Rattus norvegicus* assembly). Alignments between species-specific alternative exon 1 and/or 1c - first intron - exon 2 were performed in order to compare splice donor and acceptor sequences with the consensus splice reference sequences.

Finally, the reconstructed species-specific assembly was used as query to search, with BLASTN, the specific organism ESTs in order to prove the expression of the relative isoforms and to possibly complete the 5′ end transcript sequence.

### Accession numbers

The nucleotide sequences of identified cDNAs have been deposited in GenBank (http://www.ncbi.nlm.nih.gov/Genbank/) [36] under the following accession nos.: *RCAN3-1c,2,3,4,5* (HQ317426)*; RCAN3-1,3,4,5* (HQ287726)*; RCAN3-1c,3,4,5* (HQ317427)*; RCAN3-1c,2,4,5* (JN203053)*; RCAN3-1a,2,3,4,5* (HQ317421 and JN228252, partial coding sequence)*; RCAN3-1a,3,4,5* (HQ317422)*; RCAN3-1b,2,3,4,5* (HQ317423); *RCAN3-1b,2* (HQ317445 and HQ317446, partial coding sequence)*; RCAN3AS-1,2,3* (HQ317447 and HQ317448)*; RCAN3AS-1,3* (HQ317425), *RCAN3AS-1,2a,3* (HQ317424) and *RCAN3AS-1,2b,3* (HQ419072).

## Results

### *RCAN3* isoforms cloning and expression analysis

**Figure 1:**
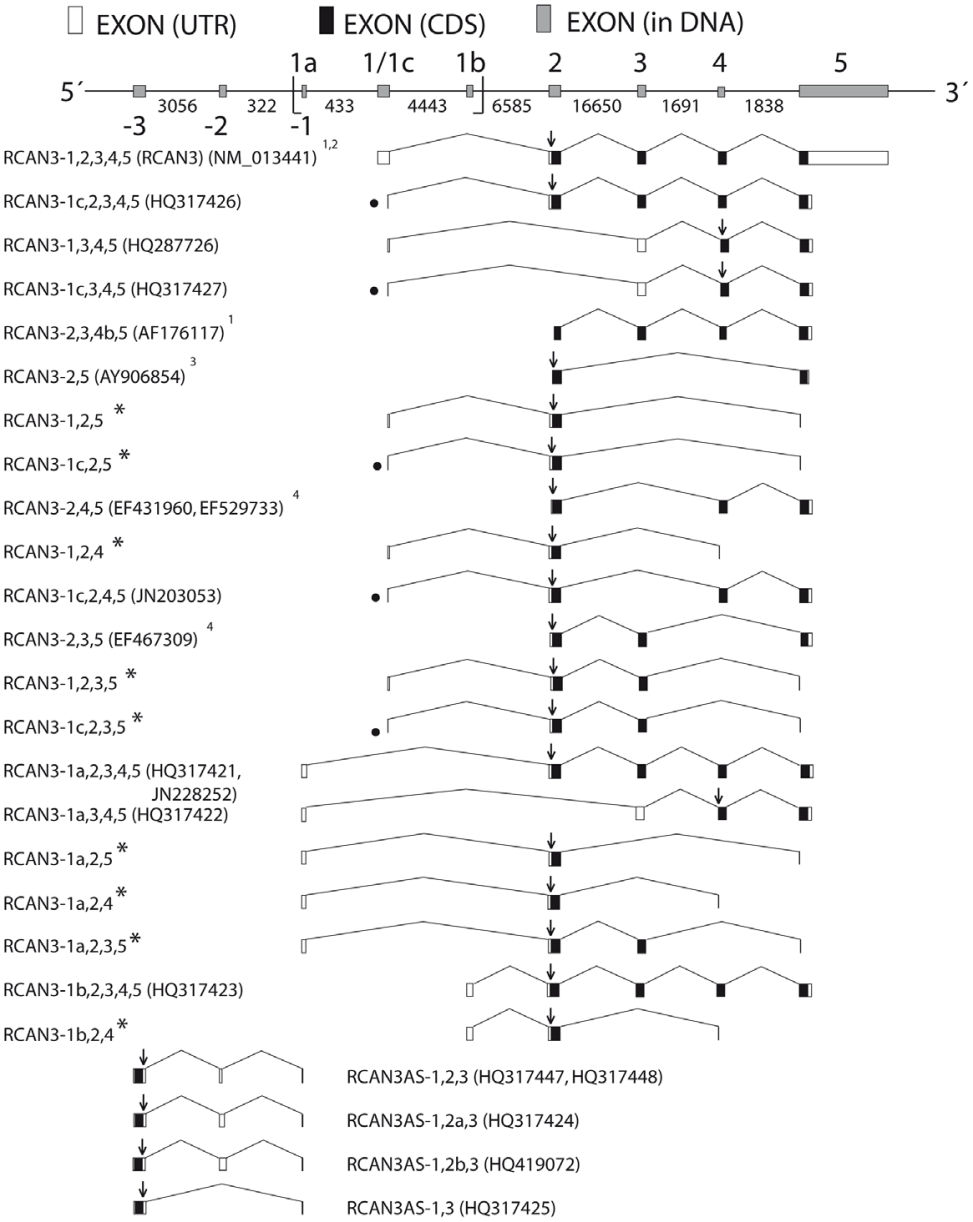
Schematic representation of *RCAN3* gene locus: *RCAN3* and *RCAN3AS* isoforms exon-intron organization. In the DNA scheme, intron sizes (represented not in scale) in base pair (bp) are indicated between the two exons; exon sizes are represented in scale, except for exon -1 (that overlaps with exon 1a on the opposite strand) and exon 1c (that lacks the last 33 bp of exon 1). Exons 1, 2 and 3 are located on the opposite strand. Exons 1 and 5 may not appear in full length in the mRNA diagram due to incompleteness of sequence determination in 5′ or 3′. The diagram, representing mRNAs, shows the longest sequences available to date (see GenBank accession numbers in the figure). In the sense transcripts the direction of translation is to the right and in the antisense transcripts it is to the left. “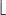 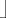
” indicates the DNA region comprised between 24,828,218 and 24,834,337 nucleotides of the chromosome 1 reference sequence NC_000001: it corresponds to the sequence showed in Figure 5A (see Figure 5A legend). ^1^ described in Strippoli et al., 2000, ^2^ described in Strausberg et al., 2002, ^3^ described in Canaider et al., 2006 and ^4^ described in Facchin et al., 2008. “*” indicates transcripts for which it was not possible to deposit a relative GenBank file. “•” indicates transcripts with exon 1c. “↓” indicates the start codon.

A combination of *in silico* and *in vitro* studies revealed several spliced *RCAN3* isoforms described below. According to the nomenclature proposed by Davies and colleagues [4], *RCAN3* mRNA isoforms are named *RCAN3*- followed by the hitherto identified exon numbers (Figure 1). The same criteria were adopted for the nomenclature of the new *Homo sapiens* gene (*RCAN3AS - RCAN3 antisense*), identified here for the first time, overlapped and on the opposite strand when compared with *RCAN3*. To give a structure as clear as possible of the “Results” section, the first three subsections below, will be identified with the name of the *RCAN3* alternative first exon that is maintained in all the isoforms described in the relative subsection. A separate subsection will be presented for *RCAN3AS*.

### *RCAN3* exon 1a

The hypothesis of the existence of an alternative exon 1 (named exon 1a) originated from the bioinformatic study of the *RCAN3* locus. A human EST sequence (GenBank accession number: BP326714, human prostate tissue source) revealed an alternative exon 1 for *RCAN3*, located on the reference genomic sequence (GenBank accession number: AL034582) 434 base pair (bp) upstream of exon 1 of the reference *RCAN3-1,2,3,4,5* isoform (NM_013441).

**Figure 2:**
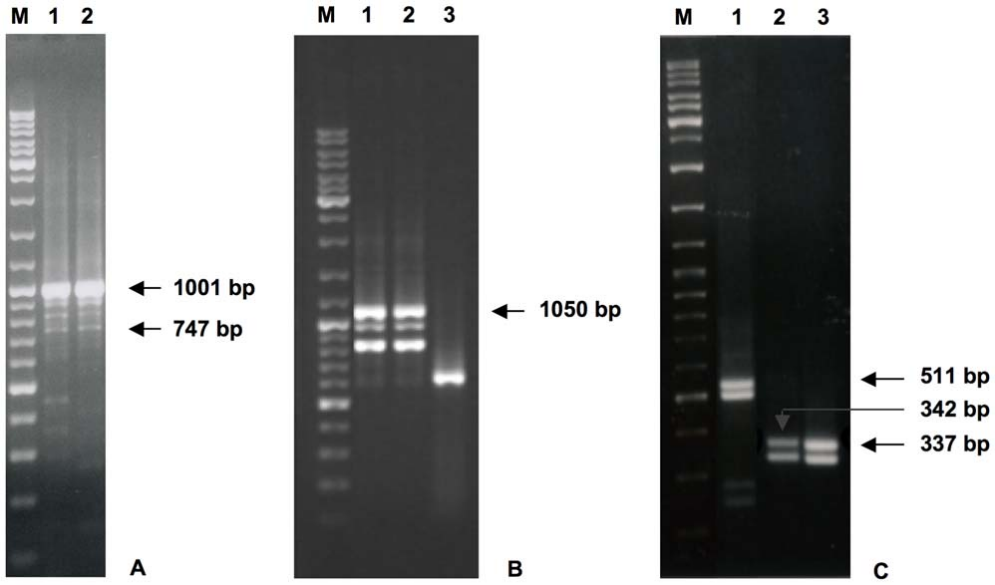
*RCAN3-1a,2,3,4,5* and *RCAN3-1b,2,3,4,5* cDNAs (example gel). A) 1% agarose gel loaded with human prostate tissue RT-PCR products. Lanes 1 and 2: *RCAN3-1a,2,3,4,5* (expected band size: 1,001 bp). The lowest band referred to the *RCAN3-1a,3,4,5* isoform (band size: 747 bp). M: size marker GeneRuler (500 ng). B) 1% agarose gel loaded with human lung tissue RT-PCR products. Lanes 1 and 2: *RCAN3-1b,2,3,4,5* (expected band size: 1,050 bp); lane 3: beta 2 microglobulin (*B2M*, expected band size: 586 bp). M: size marker GeneRuler (500 ng). Other bands than the expected and not indicated are referred to RT-PCR artifacts or to *RCAN3-1a,2,4*, *RCAN3-1a,2,5*, *RCAN3-1a,2,3,*5 (Figure 2A) and *RCAN3-1b,2,4* (Figure 2B) transcript assemblies whose existences were demonstrated by RT-PCRs performed with specific primers, as described in the text. C) 1% agarose gel loaded with human peripheral blood leucocytes RT-PCR products. Lane 1: *RCAN3-1,2,3,5* (expected band size: 511 bp) and *RCAN3-1c,2,3,5* (unexpected band size: 478 bp); lane 2: *RCAN3-1,2,5* (expected band size: 342 bp) and *RCAN3-1c,2,5* (unexpected band size: 309 bp); lane 3: *RCAN3-1,2,4* (expected band size: 337 bp) and *RCAN3-1c,2,4* (unexpected band size: 304 bp). M: size marker GeneRuler (500 ng).

Therefore, a first pair of primers (#3 and #4, Table 1) was designed on the most external sequence of the #BP326714 EST. Sequence analysis of the RT-PCR product demonstrated the existence of an mRNA sequence in human prostate (*RCAN3-1a,2,3,4*, GenBank accession number: GQ411200), as reported and discussed in our recent paper [19]. A new reverse primer (# 2, Table 1) was then designed on the exon 5, in order to elongate the sequence. Gel analysis revealed more than one PCR product (Figure 2A) and sequence analysis showed the existence of two new alternative *RCAN3* isoforms: the expected new longer spliced sequence *RCAN3-1a,2,3,4,5* (GenBank accession number: HQ317421) and a spliced alternative isoform lacking exon 2, named *RCAN3-1a,3,4,5* (GenBank accession number: HQ317422). Sequence analyses also revealed the presence of possible RT-PCR artifacts, due to the presence of distant sequence repetition, which might induce a tridimensional RNA structure formation that prevents the normal RT-PCR process [37]. The existence of the *RCAN3-1a,3,4,5* transcript was confirmed by an RT-PCR amplification using *RCAN3* isoform specific primers (#17 and #2, Table 1). *RCAN3-1a,2,3,4,5* gene comprises five exons, with the alternative first exon (exon 1a) (Figure 1). Moreover, RT-PCR performed with *RCAN3-1a* isoform specific forward and reverse primers demonstrated the existence of three additional transcripts (*RCAN3-1a,2,4*, *RCAN3-1a,2,5*, *RCAN3-1a,2,3,5*), which were not revealed by the sequence analyses described above (Figure 1).

RACE assay revealed the existence of an *RCAN3-1a,2,3,4,5* transcript (GenBank accession number: JN228252, Figure 1) with additional 19 bp at the 5′ end, compared with the *RCAN3-1a,2,3,4,5* sequence previously deposited (#HQ317421). In addition, the obtained 5′ end is coherent with the reference EST sequence (#BP326714), compared to which lacks the first 5 bp and identifies a real TSS (transcription start site) of the *RCAN3-1a,2,3,4,5* transcript, within a putative transcription start region.

An expression panel of *RCAN3-1a,2,3,4,5* cDNAs was obtained by RT-PCR (primer #3 and #4, Table 1) from total RNAs extracted from 17 adult human whole normal tissues (see “RT-PCR expression analysis” subsection for tissues choice, data not shown). In all tissues, gel electrophoresis analysis revealed the expected size band referred to *RCAN3-1a,2,3,4,5*. Occasionally, two other products of unexpected size were observed, one of which is referred to the *RCAN3-1a,3,4,5* spliced isoform. *RCAN3-1a,2,3,4,5* seems to be clearly expressed in prostate tissue, in agreement with the reference EST source.

### *RCAN3* exon 1b

The hypothesis of the existence of exon 1b also originated from the bioinformatic study of the *RCAN3* locus. A human EST sequence (GenBank accession number: CD700433, human nasopharynx tissue source) revealed an alternative exon 1 for *RCAN3*, located on the reference genomic sequence (#AL034582) 4,443 bp downstream of exon 1 of the reference *RCAN3* isoform (#NM_013441). Therefore, two primers (#5 and #6, Table 1) were designed on the most external sequence of the #CD700433 EST. Sequence analysis of the RT-PCR product demonstrated the existence of an mRNA sequence in human lung and testis (GenBank accession numbers: HQ317445 and HQ317446, respectively), matching the exon - exon junction between the new exon 1b and the exon 2 of *RCAN3*. The reverse primer # 2 (Table 1), designed on exon 5, was used to elongate the sequence. Thus, a new longer sequence was cloned in lung tissue (GenBank accession number: HQ317423) (Figure 2B). *RCAN3-1b,2,3,4,5* mRNA consists of five exons, where the first one (exon 1b) is alternative to exon 1 of *RCAN3-1,2,3,4,5* (Figure 1). Sequence analysis of PCR products revealed the existence of other sequences due to RT-PCR artifacts as previously discussed for *RCAN3-1a,2,3,4,5* [37]. To verify the *RCAN3-1b,3,4,5* transcript absence an RT-PCR amplification with isoform specific primers (#18 and #2, Table 1) was performed, thus confirming previous results, unlike what was observed for the other isoforms lacking exon 2 with alternative first exons 1, 1c and 1a. Moreover, RT-PCR performed with *RCAN3-1b* isoform specific forward and reverse primers demonstrated the existence of one additional transcript (*RCAN3-1b,2,4*), which was not revealed by the sequence analyses described above (Figure 1).

With regard to *RCAN3-1a,2,3,4,5*, an expression panel of the same 17 normal human tissues (see “RT-PCR expression analysis” subsection for tissues choice, data not shown) was obtained for *RCAN3-1b,2,3,4,5* (primers #5 and #23). In all tissues, gel electrophoresis analysis revealed one product of the expected size. Expression appears to be very low in many tissues, except for lung where it is more evident.

### *RCAN3* exon 1 and exon 1c

Exon 1 is referred to the exon of reference for the *RCAN3* isoform (#NM_013441), while exon 1c is a new exon identified here for the first time, together with exon 1a and exon 1b, and that corresponds to the exon 1 lacking the last 33 bp.

RT-PCR (using primers #1 and #2) was performed on human peripheral blood leukocytes cDNA to obtain *RCAN3* cDNA in order to identify new alternatively spliced *RCAN3* isoforms and to 5′ elongate previously identified *RCAN3* isoforms (*RCAN3-2,3,4b,5*, *RCAN3-2,4,5*, *RCAN3-2,3,5* and *RCAN3-2,5*), assuming the presence of reference exon 1 (Figure 1). Gel analysis revealed more than one PCR product. In fact, RT-PCR products unexpectedly proved to contain three new *RCAN3* spliced isoforms in addition to the known full length *RCAN3* cDNA (GenBank accession numbers: NM_013441 and AF176116): *RCAN3-1c,2,3,4,5* cDNA (GenBank accession number: HQ317426), lacking the last 33 bp of exon 1, *RCAN3-1,3,4,5* cDNA (GenBank accession number: HQ287726), lacking exon 2, and *RCAN3-1c,3,4,5* cDNA (GenBank accession number: HQ317427), lacking exon 2 and the last 33 bp of exon 1 (Figure 1). The existence of the *RCAN3-1,3,4,5* and *RCAN3-1c,3,4,5* isoforms were confirmed by RT-PCR amplification using *RCAN3* specific forward primers (Table 1, #15 and #16, respectively) and a common reverse primer (#2, Table 1).

Sequence analysis did not reveal any sequence relating to the four previously identified *RCAN3* isoforms. Only one product relating to exon 2 - exon 4 - exon 5 and containing the exon 1c was retrieved (Figure 1). The presence of an EST corresponding to this exon - exon junction (#CT001954) confirmed the existence of the *RCAN3-1c,2,4,5* isoform. Due to the presence of two nucleotide variants in our product, with respect to the EST and genomic sequences, and the impossibility to refer them to known SNP (single nucleotide polymorphism) clusters, we submitted a corresponding GenBank file (GenBank accession number: JN203053, Figure 1) whose sequence presents two “N” (76 and 331 nucleotide positions), in order to indicate the difficulty to identify with certainty the corresponding nucleotides.

Further specific reverse primers (Table 1: #9, #10, #11, #12) were designed to demonstrate the presence of exon 1 in the *RCAN3-2,3,4b,5*, *RCAN3-2,5*, *RCAN3-2,4,5* and *RCAN3-2,3,5* isoforms.

In all RT-PCR, gel analysis revealed two PCR products (Figure 2C). Sequence analysis revealed that they corresponded to the isoforms *RCAN3-1,2,4* and *RCAN3-1c,2,4*, *RCAN3-1,2,3,5* and *RCAN3-1c,2,3,5* or *RCAN3-1,2,5* and *RCAN3-1c,2,5* (Figure 1). Since the reverse primers were designed encompassing specific exon - exon boundaries and having to remove the primer sequence from the cloned sequence, we could not deposit the corresponding GenBank files. Sequence analysis of *RCAN3-2,3,4b,5* RT-PCR products revealed the presence of many nucleotide variants, which did not allow us to refer them to specific isoforms.

**Figure 3:**
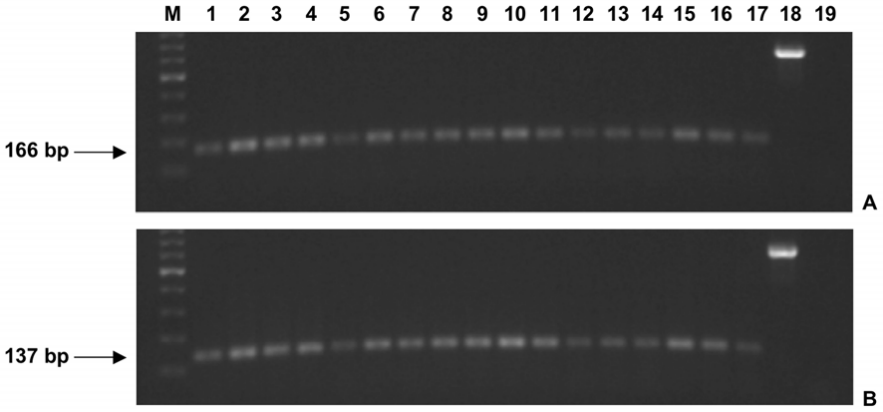
Representative expression panel of *RCAN3* exon 1 and exon 1c (example gel). 1% agarose gel loaded with human normal tissues RT-PCR products: exon 1 - exon 2 junction cDNA in A and exon 1c - exon 2 junction cDNA in B (expected band size: 166 bp and 137 bp, respectively). Lanes: 1, brain; 2, prostate; 3, peripheral blood leukocytes; 4, thymus; 5, spleen; 6, stomach; 7, colon; 8, bone marrow; 9, testis; 10, lung; 11, small intestine; 12, adrenal gland; 13, mammary gland; 14, liver; 15, pancreas; 16, heart; 17, skeletal muscle. M: size marker GeneRuler (500 ng), lane 18: B2M (expected band height: 586 bp) in peripheral blood leukocytes; lane 19: negative control.

Further primer pairs were designed to verify the expression of all isoforms containing exon 1 - exon 2 junction (#24 and #26, Table 1) and of all isoforms containing exon 1c - exon 2 junction (#25 and #26, Table 1) in 17 normal human tissues (see “RT-PCR expression analysis” subsection). In all cases, gel electrophoresis analysis revealed one product of the expected size for all analyzed tissues. Since the same expression pattern is evident (Figure 3A and B), it is conceivable that the alternative use of exon 1 or exon 1c is stochastic [38].

### *RCAN3AS*

**Figure 4:**
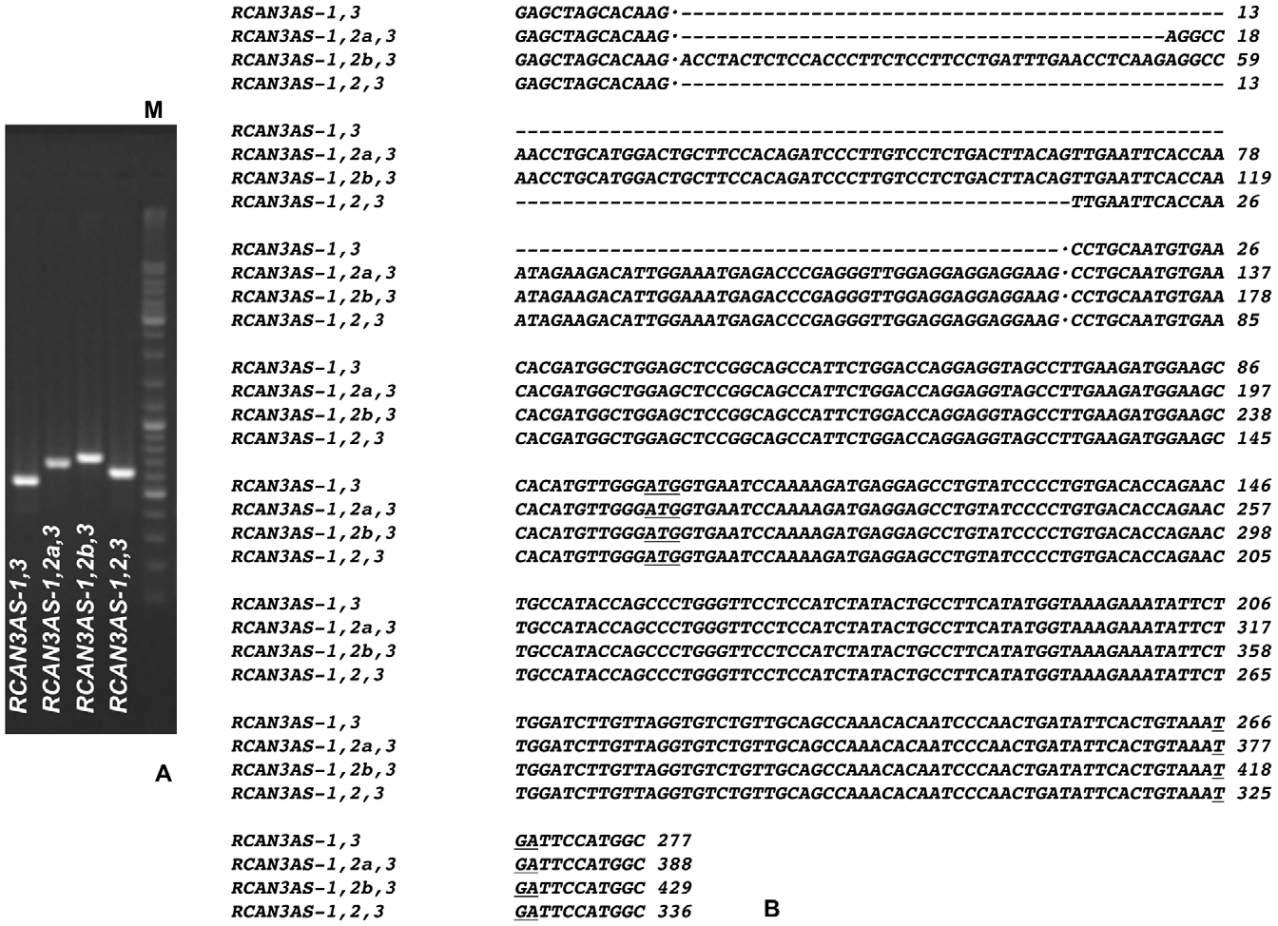
*RCAN3AS* mRNA isoforms sequence identification and analysis. A) 1% agarose gel loaded with *RCAN3AS* colony PCR products. From left to right: *RCAN3AS-1,3* (expected band size: 547 bp), *RCAN3AS-1,2a,3* (expected band size: 658 bp), *RCAN3AS-1,2b,3* (expected band size: 699 bp) and *RCAN3AS-1,2,3* (expected band size: 606 bp). M: size marker GeneRuler (500 ng). Band heights result by using specific pCR2.1 plasmid primer pairs. B) *RCAN3AS* mRNA isoform sequences aligned by ClustalW software. Exon 2a and exon 2b are longer than exon 2 in their 5′ (52 bp and 93 bp, respectively).

Since the bioinformatic analysis revealed one EST (GenBank accession number: BF448186) on the complementary strand and overlapping *RCAN3*, primers (#7 and #8, Table 1) were designed to amplify this cDNA from testis and pancreas RNAs. RT-PCR products revealed more than one band on agarose gel. All PCR products were cloned in pCR2.1 plasmid and the sequences of several transformed clones were determined. Four different isoforms were identified (Figure 4A) and their denomination has recently been approved by the HUGO Gene Nomenclature Committee. *RCAN3AS* name indicates that this gene is on the opposite strand compared to *RCAN3* and *RCAN3AS* isoforms were named on the basis of the exon organization. We considered *RCAN3AS-1,2,3* (GenBank accession numbers: HQ317447 - testis and HQ317448 - pancreas, respectively) as reference isoform since it matches the #BF448186 EST. It consists of 3 exons, it is located on the *RCAN3* locus (1p36.11) and it is on the opposite strand of *RCAN3* (Figure 1). Moreover, *RCAN3AS-1,2,3* overlaps with the first 32 bases of the *RCAN3* exon 1a, according to the two corresponding EST sequences.

The other three unexpected *RCAN3AS* isoforms have been cloned by RT-PCR in human pancreas: *RCAN3AS-1,2a,3* (GenBank accession number: HQ317424), *RCAN3-1,2b,3* (GenBank accession number: HQ419072) and *RCAN3AS-1,3* (GenBank accession number: HQ317425) (Figure 1). *RCAN3AS-1,2a,3* and *RCAN3AS-1,2b,3* consist of 3 exons, with an elongation of 52 bp and of 93 bp of the exon 2 (in 5′), respectively, compared with exon 2 of *RCAN3AS-1,2,3* (Figure 4A and B). *RCAN3AS-1,3* consists of 2 exons, since it lacks exon 2 completely, compared with the *RCAN3AS-1,2,3* reference sequence (Figure 4A and B).

To obtain preliminary information about *RCAN3AS-1,2,3* expression, RT-PCR with primers #7 and #8 (Table 1) was performed in 17 adult human whole normal tissues (see “RT-PCR expression analysis” subsection for tissues choice, data not shown). In all cases, gel electrophoresis analysis revealed the presence of the expected size band referred to *RCAN3AS-1,2,3*, which is present in all investigated tissues and in the same conditions of amplifications it seems to be more expressed in testis and pancreas.

### Bioinformatic analysis of *RCAN3* mRNA isoforms

To verify if alternative *RCAN3* isoforms were present in human sequence databases, a search at NCBI databases was performed by BLASTN software.

Human EST sequences matching alternative first exon - exon 2 junction were identified and assigned to a specific isoform after manual analysis of the retrieved sequences. Human EST sequences matching alternative first exon - exon 3 junction were not identified. Detailed summary of results obtained by this bioinformatic analysis is described in Table 2. Many ESTs extending from exon 2 to subsequent exons were found, but due to the high complexity of the *RCAN3* locus, they could be assigned to any isoform containing these exons and any alternative first exon and they were not compared in Table 2.

**Table 2:** *RCAN3* cDNA isoforms in *Homo sapiens* databases.

cDNA	*Homo sapiens* a nr/nt or ref_seq	*Homo sapiens* EST	EST tissue or cells
*RCAN3-1,2,3,4,5*	NM_013441 BC035854	BM928218	Brain
		CR986931	Uncharacterized tissue
		DN991656	Brain
		BU180267	Pancreas
		BU157485	Pancreas
		BQ894646	Pancreas
		BQ687884	Pancreas
		BQ686955	Pancreas
*RCAN3-1,2,4,5*	None	BM808737	Hippocampus
*RCAN3-1,3,4,5*	None	None	
*RCAN3-1a,2,3,4,5*	GQ411200	BP326714	Prostate
		AW408648l	Lymph, germinal center B cells
*RCAN3-1a,3,4,5*	None	None	
*RCAN3-1b,2,3,4,5*	None	CD700433	Nasopharynx
*RCAN3-1c,2,3,4,5*	None	BQ929181	Prostate
		CX752875	Cell line derived from blastocyst
		BX642220*	Not declared
*RCAN3-1c,2,4,5*	None	CT001954	T-Lymphocytes
*RCAN3-1c,3,4,5*	None	None	

Search was performed with BLASTN software (default parameters, with no filter).

Alternative first exon - exon 2 or 3 query sequences were used. Manual analysis of entire found sequences allowed us to assign them to a specific isoform.

a excluding genomic sequences. “nr” (non redundant), “nt” (nucleotide), “ref_seq” (reference sequence) and “EST” (expressed sequence tag).

*This EST extends only up to exon 3.

The same results were obtained using the ECgene Browser and the UCSC Genome Browser. These two independent browsers showed another alternative isoform (GenBank accession number: BU947377), which was not further analyzed in the present work. BU947377 EST showed exons 3-4-5 linked to a new alternative exon 2. Moreover, an analysis of newly identified *RCAN3* mRNA sequences was performed. Three different SNPs for the *RCAN3* mRNA isoform have been reported in the single nucleotide polymorphism database (dbSNP) at the NCBI (http://www.ncbi.nlm.nih.gov/entrez/query.fcgi?db=snp) [39] as clusters rs196429 (average heterozygosity ± standard error: 0.428±0.175), rs196430 and rs196432 (average heterozygosity ± standard error: 0.497±0.040), as discussed by Facchin et al. [22]. The first two SNPs were found in exon 4, while the third was found in exon 5 (nucleotide positions 727 bp, 775 bp and 976 bp, respectively, referring to the #NM_013441 and #BC035854 sequences). The presence of G or A in all three SNPs described does not change the encoded amino acid sequence (amino acids 138 and 154 are proline and 221 is threonine, respectively, referring to NM_013441). In Table S1, the presence of G or A are reported for all 6 newly identified *RCAN3* isoforms (*RCAN3-1,3,4,5*, *RCAN3-1c,2,3,4,5*, *RCAN3-1c,3,4,5*, *RCAN3-1a,2,3,4,5*, *RCAN3-1a,3,4,5* and *RCAN3-1b,2,3,4,5*), on the basis of the retrieved nucleotide in the corresponding position of our cloned isoforms (“GenBank accession no.”, second column of Table S1). SNPs data referred to previously described *RCAN3* isoforms were discussed in Facchin et al. [22].

Search by BLASTN software in nr/nt, refseq_rna and EST databases at NCBI in all organisms (excluding *Homo sapiens*) identified only sequences similar to human exon 1 - exon 2 and human exon 1c - exon 2 junctions, which were assigned to *RCAN3-1,2,3,4,5* and *RCAN3-1c,2,3,4,5* after manual analysis of the entire sequences retrieved (Table S2). No defined or reference sequence or EST transcript matching human exon 1 - exon 3, exon 1a - exon 2, exon 1a - exon 3 or exon 1c - exon 3 junctions were identified, except for one *Sus scrofa* EST sequence matching human exon 1c - exon 3 (GenBank accession number: BW970272). The BLASTN search performed using the human alternative first exons - exon 2 or 3 sequences as query against all the genomic databases, lead to the observation, in some organisms, of the presence of sequences matching with human first exons (Table S2 and Table S3), allowing us to speculate on the existence of relative species-specific transcripts containing them.

Comparing splice donor sequences surrounding GT signal site in the transition between alternative first exons and following first intron with the consensus reference sequence (CAGGTRAGT, where R is referred to a purine nucleotide – [32]), exon 1c donor sequence results to be the more adherent. Splice donor sequences of exon 1, 1a and 1b showed 1, 2 and 3 nucleotides substitution (purine/purine or pyrimidine/pyrimidine), respectively, when compared with the consensus sequence. Splice donor sequences of exon 1 showed 2 nucleotide differences, while exon 1c and exon 1a showed one difference. Comparing splice acceptor sequences surrounding AG signal site in the transition between alternative first introns and exon 2 or 3 with the consensus reference sequence (YACN, where Y is referred to pyrimidine nucleotide and N to any nucleotide – [32]), all studied acceptor sequences were conserved (Figure S1). In figure S1, the exon 1b - first intron - exon 3 sequence is not shown, since we have not retrieved a corresponding mRNA.

### *RCAN3* promoter analysis

**Figure 5:**
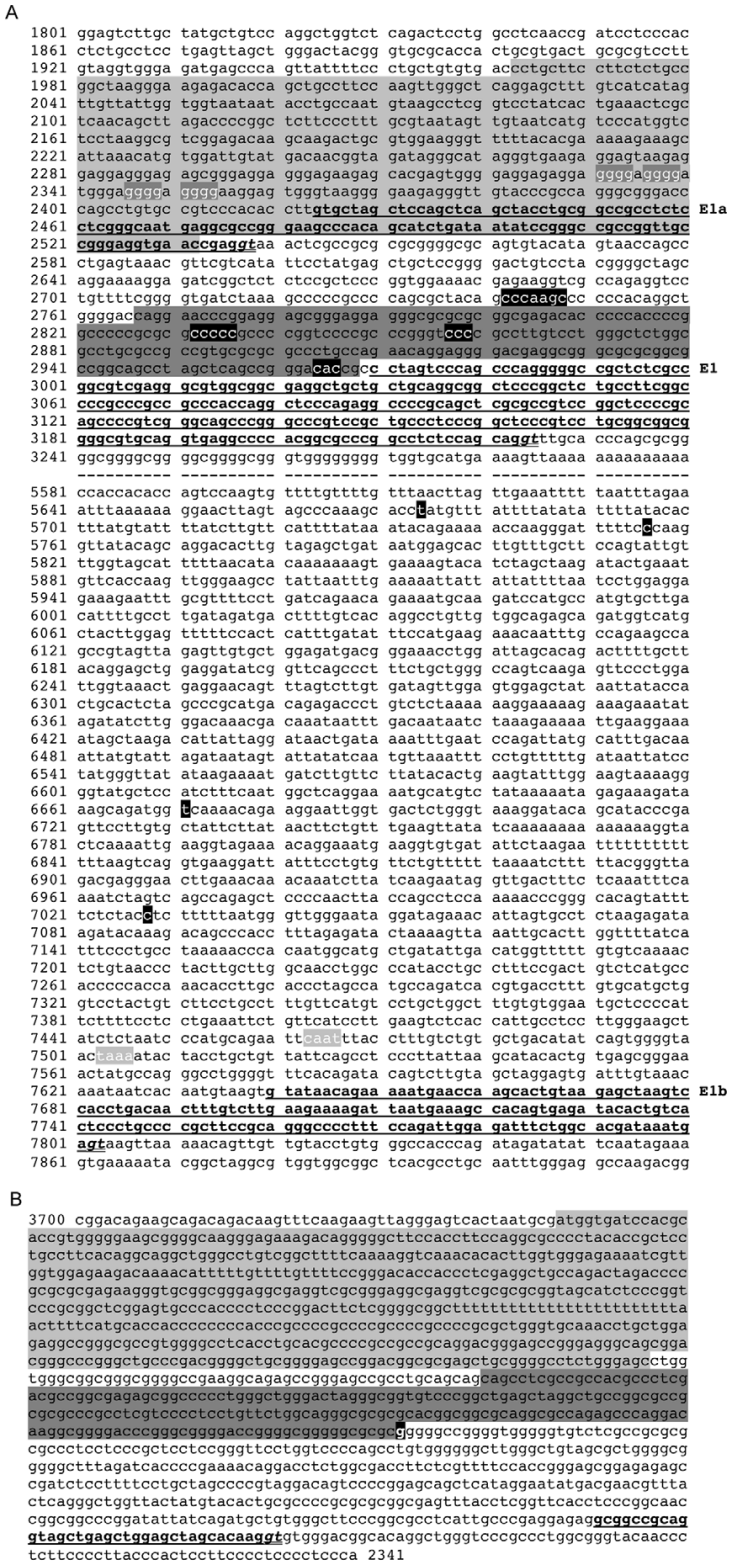
Promoter analysis in *RCAN3* alternative first exons and RCAN3AS isoforms. Highlighted in light grey: promoter region; highlighted in dark grey: CpG island; in bold and underlined: exon sequence; in bold, italic and double underlined: GT splice signal; in bold, white and highlighted in black: transcription start site (TSS). A) AL034582 genomic sequence from 1,801 to 7,920 nucleotides, corresponding to the chromosome 1 reference sequence NC_000001 from 24,828,218 to 24,834,337 nucleotides, respectively. The same genomic interval is indicated by two graphic symbols in the DNA scheme of Figure 1. In bold, white and highlighted in dark grey: guanine repetitions; in bold, white and highlighted in light grey: “CAAT box” and “TATA box”; - -: genomic interval. B) AL034582 reverse genomic sequence (1-1,360 bp corresponding to 3,700-2,341 bp of AL034582 direct sequence) with *RCAN3AS* exon 1 (bold and underlined).

Promoter analysis in order to detect consensus promoter sequences and hypothetical TSSs was performed on AL034582 as genomic reference sequence (Figure 5A).

Only one CpG island of 202 bp in length was found by First Exon Finder software upstream exon 1 of *RCAN3* gene, extending from nucleotide (nt) 2,767 to nt 2,968 and with an high GC percentage/content (GC 82%) (the *RCAN3* mRNA begins at nt 2,970 and exon 1 ends at nt 3,223). Using the same software, no CpG island was detected adjacent to exon 1a or exon 1b. Three short sequences containing possible TSSs were identified by Promoter Prediction tool (Berkeley Drosophila Genome Project) and by Neural Network Promoter Prediction server within the CpG island (2,832-2,836, 2,857-2,859 and 2,964–2,966 nt positions, according to exon 1 boundaries). Another short sequence with TSSs was identified upstream the CpG island (2,742–2,748 nt positions). The identified short sequence at 2,964–2,966 nucleotide positions is very close to the first nucleotide of the NM_013441 reference transcript, assembled considering the complete 5′ *RCAN3* sequence derived from a massive sequencing project whose purpose was to clone full human ORFs (open reading frames) from libraries enriched for full-length cDNAs [24]. Therefore, the identified sequence containing a possible TSS could correspond to a real transcription start region.

First Exon Finder software detected a promoter region from nt 1,963 to nt 2,532, a region starting upstream of exon 1a and ending within the same exon (the *RCAN3-1a,2,3,4,5* mRNA begins at nt 2,424 and its exon 1 ends at nt 2,536, according to BP326714 reference EST), whose possible TSS has been verified by RACE method (as described in subsection “*RCAN3* exon 1a”). Moreover, manual analysis of genomic sequence upstream exon 1a revealed a guanine repetition responsible for G-quadruplex structure formation [40]. The regulated formation of these structures in the promoter region has been demonstrated to provide an elegant nucleic-acid-based mechanism for transcription modulation [41].

Four TSSs were also predicted upstream of exon 1b (from nt 7,640 to nt 7,801, according to CD700433 reference EST) at 5,674, 5,756, 6,671 and 7,028 nucleotide positions by Promoter Prediction tool (Berkeley Drosophila Genome Project). However, no TSS lied adjacent the known exon 1b start. Given the average length of 210 bp of human genes 5′UTR (18 bp and 2858 bp, minimum and maximum 5′UTR length, respectively) [42], only the TSS located in position 7,028 may be plausible, also assuming that exon 1b could extend upstream [42], [43].

On the other hand, manual analysis of genomic sequence (16 bp and 177 bp upstream, respectively) of exon 1b revealed a possible “TATA box” (TAAA from nt 7,503 to nt 7,506) and a “CAAT box” (CAAT from nt 7,463 to nt 7,466), that no Promoter Prediction software had identified. The distance between the two sequence boxes is coherent.

### RCAN3 protein isoforms

**Figure 6:**
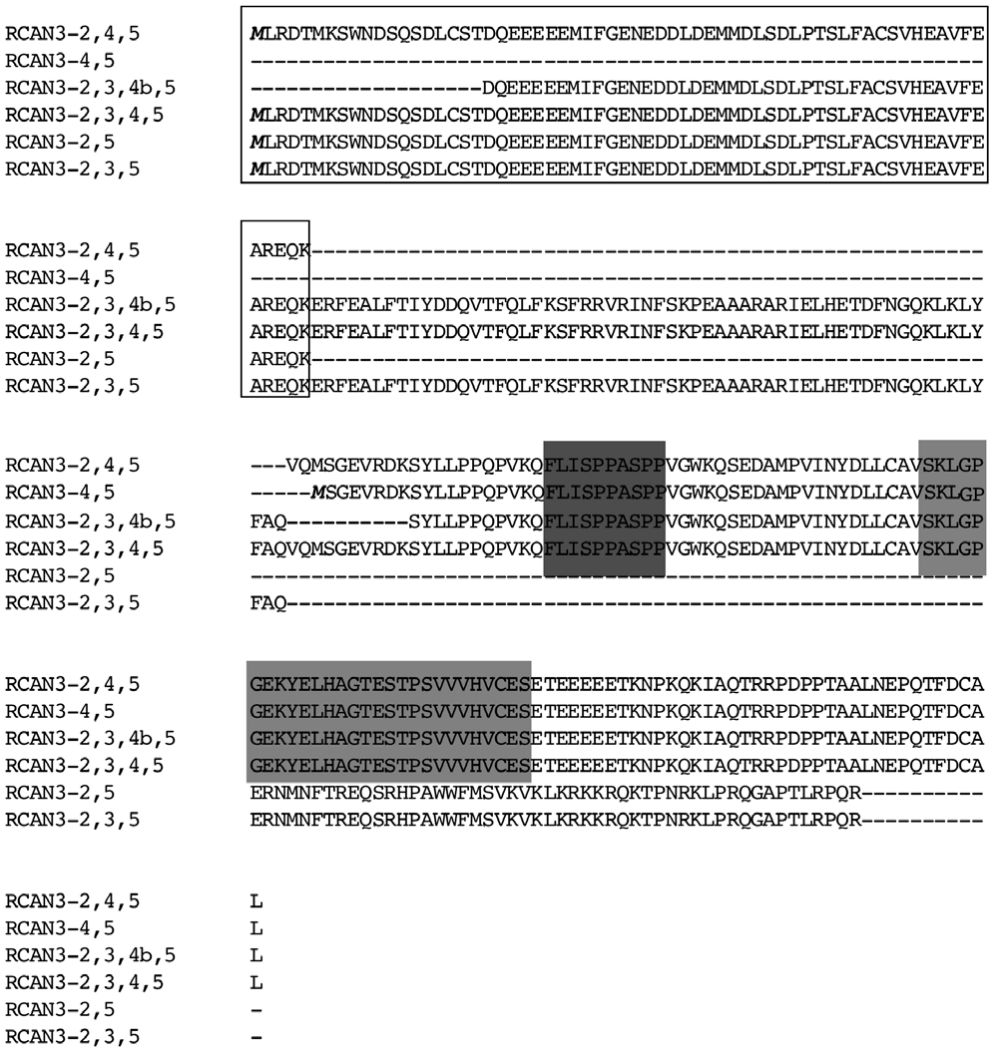
RCAN3 isoform predicted protein sequences aligned by ClustalW software. RCAN3-2,3,4,5 here refers to *RCAN3-1,2,3,4,5, RCAN3-1a,2,3,4,5, RCAN3-1b,2,3,4,5* and *RCAN3-1c,2,3,4,5* mRNA products. RCAN3-2,4,5 refers with certainty to *RCAN3-1c,2,4,5* mRNA product. In all these isoforms the coding sequence starts in exon 2 and finishes in exon 5. RCAN3-4,5 refers to *RCAN3-1,3,4,5, RCAN3-1a,3,4,5* and *RCAN3-1c,3,4,5* product sequences. In all these isoforms the coding sequence starts in exon 4. Due to the lack of exon 2, RCAN3-4,5 sequence is shorter than RCAN3-1,2,3,4,5 protein and lacking the amino terminus containing the exon 2 product necessary for human cardiac troponin I (TNNI3) binding. Due to out-of-frame joining between exons 2 and 5 and between exons 3 and 5, the carboxyl terminus sequence encoded by *RCAN3-2,5* and by *RCAN3-2,3,5* cDNAs, respectively, is not similar to that of *RCAN3-1,2,3,4,5* product sequence. In bold and italic the initial methionine of the proteins. Black border box: exon 2 encoded amino acids; shaded dark grey residues: FLISPP motif. Shaded light grey: CIC motif, with the embedded ELHA motif (KYELHAGTESTPS). The diagram shows the longest protein sequences available to date (see mRNA GenBank accession numbers in text or figure 1). Alignment was made by ClustalW software and manual adjustment.

Figure 6 shows the alignment (performed by ClustalW software and manually revised) of the 6 human RCAN3 protein isoform sequences (RCAN3-2,3,4,5, RCAN3-2,4,5, RCAN3-2,3,4b,5, RCAN3-2,3,5, RCAN3-2,5 and RCAN3-4,5). RCAN3-2,3,4,5 protein is referred to *RCAN3-1,2,3,4,5*, *RCAN3-1a,2,3,4,5, RCAN3-1b,2,3,4,5* and *RCAN3-1c,2,3,4,5* mRNA products (RCAN3 reference protein, GenBank accession number: NP_038469). In these mRNA isoforms the conserved coding sequence starts in exon 2 and finishes in exon 5. RCAN3-2,4,5 refers with certainty to *RCAN3-1c,2,4,5* mRNA product, where frame is conserved in junction between exons 2 and 4. RCAN3-4,5 is the product of *RCAN3-1,3,4,5*, *RCAN3-1a,3,4,5,* and *RCAN3-1c,3,4,5* mRNAs, whose coding sequence starts in exon 4. Alignment analysis highlights that the exon 2 encoded domain is the same in all the alternative RCAN3 isoforms that contain it. The product of exon 2 has been shown to be sufficient for binding to TNNI3 [20]. All the studied proteins, with the exception of RCAN3-2,3,5 and RCAN3-2,5, contain the CIC motif, the calcineurin binding site [7]. Due to the out-of-frame joining between exons 3 and 5 and between exons 2 and 5, the carboxyl terminus sequence encoded by *RCAN3-2,3,5* and by *RCAN3-2,5* cDNAs is not similar to that of the RCAN3 product sequence, as previously reported by us [22].

All these protein sequences do not present similarities with any other human putative domain as searched by the BLASTP software (default parameters, with no filter).

### Comparative sequence analysis in all organisms

NM_013441 (human *RCAN3-1,2,3,4,5* gene) and NP_038469 (human RCAN3-1,2,3,4,5 protein) were used as initial query sequences to retrieve all transcripts relative to other organisms by BLASTN and TBLASTN software in nr/nt database at NCBI. With regard to *RCAN3-1,2,3,4,5*, transcript models were found only in primates. On the contrary, transcribed sequences and transcript models referred to *RCAN3-1c,2,3,4,5* were found in primates, mammals - not primates, and not mammals (Table S3, second column). For all identified transcripts, the corresponding reference genomic sequences were retrieved (Table S3, third column). The analyses described in this paper allowed us to observe more transcript sequences and relative genomes compared to those indicated in Table S3 (retrieved sequences also for *Equus caballus*, *Oryctolagus cuniculus*, *Ornithorhynchus anatinus*, *Monodelphis domestica*, *Taeniopygia guttata* and *Xenopus tropicalis*). However, they were not reported because incomplete and insufficient to rebuild exon1/1c - first intron - exon 2 junctions. Figure S2 shows species-specific alignments relative to exon 1 and/or exon 1c - first intron - exon 2 in all organisms, identified by bioinformatic search and following manual adjustment. The study of the donor splicing sequences for all studied genomic sequences showed the presence of high sequence conservation compared to human consensus sequence, especially when it is referred to the exon 1c - exon 2 junction. Moreover, only in primates (white block in Figure S2) both donor signal sites were present on genomic sequences. The donor splice sequence related to exon 1c showed an identity of 89% with consensus sequence, while the donor splice sequence related to exon 1 showed an identity of 67%. The conservation of both splice donor sequences suggests their alternative possible use as it happens in *Homo sapiens*. In mammals-not primates (light grey block in Figure S2), the comparison of genomic sequences showed high conservation of sequences compared to human genomic sequences and only the donor splice site, corresponding to human exon 1c, was retrieved (donor splice sequences showed identity of 100% with the consensus sequence, 9/9 bp). The splice donor site related to human exon 1 was not present. Even in not mammal organisms (*Gallus gallus* and *Danio rerio*), the analysis of genomic sequences and the absence of transcribed sequences exon 1 - like indicated that only the splice site similar to human exon 1c could be used, whose signal sequences are highly conserved (dark grey block in Figure S2).

Finally, a species-specific exon 1 - exon 2 or exon 1c - exon 2 assembly were used as query to search by BLASTN the specific organism ESTs, in order to prove the expression of the relative isoforms and to complete the 5′ end transcript sequence. No ESTs related to the presence of human exon 1 - exon 2 junction were identified. ESTs referred to exon 1c - exon 2 junction were retrieved for *Bos taurus*, *Sus scrofa*, *Canis lupus familiaris*, *Mus musculus*, *Gallus gallus* and *Danio rerio* (Table S3, fourth column).

### Bioinformatic analysis of *RCAN3AS* mRNA isoforms

In order to verify if *RCAN3AS* isoforms were present in human sequence databases, a search of NCBI databases was performed by BLASTN software. For all *RCAN3AS* isoforms reference genomic sequences were represented by the two contigs AL031431 and AL034582. Table 3 resumed EST identified sequences and their tissue source.

**Table 3:** *RCAN3AS* cDNA isoforms in *Homo sapiens.*

cDNA	*Homo sapiens* nr/nt or ref_seq	*Homo sapiens* EST	EST tissue
*RCAN3AS-1,2,3*	AL031431 AL034582	BF448186	Lung carcinoid
		AW339253	Lung
		BG149353*	Lung carcinoid
		AW339444*	Lung carcinoid
		AW134996*	Lung carcinoid
		AA992107*	Testis
*RCAN3AS-1,2a,3*	AL031431 AL034582	BQ025862	Placenta
		BE551182	Lung carcinoid
		BE551149	Lung carcinoid
*RCAN3AS-1,2b,3*	AL031431 AL034582	CR741819	Testis
*RCAN3AS-1,3*	AL031431 AL034582	None	None

Search was performed by BLASTN software (default parameters, with no filter).

“nr” (non redundant), “nt” (nucleotide), “ref_seq” (reference sequence) and “EST” (expressed sequence tag).

*ESTs that could be referred to any *RCAN3AS* alternative exon 2 isoforms.

The same EST results were obtained for the four *RCAN3AS* isoforms using the ECgene Browser and the UCSC Genome Browser. These two independent browsers showed another alternative isoform (GenBank accession number: BM457392), which was not further analyzed in the present work. BM457392 was an expressed sequence on the opposite strand of *RCAN3* and had three exons. The third exon is overlapping but more extended in 3′ compared to *RCAN3AS* exon 3.

Search by BLASTN software in nr/nt, refseq_rna and EST databases at NCBI in all organisms (excluding *Homo sapiens*) did not identify sequences similar to human *RCAN3AS* isoforms.

Analysis of the splice donor sequence surrounding the GT signal site in the transition between exon 1 and the first intron of RCAN3AS isoforms with the consensus reference sequence underlined a high similarity. Comparing specific splice acceptor sequences surrounding the AG signal site in the transition between first introns and alternative exons 2 (2, 2a, 2b) or exon 3 and the consensus reference sequence, we observed that all studied acceptor sequences resulted very adherent, except for a nucleotide difference in the first intron - exon 2a boundary (Figure S3).

### *RCAN3AS* promoter analysis

In order to detect consensus promoter sequences and a hypothetical TSS, promoter analysis was performed by using AL034582 as genomic reference sequence (Figure 5B). A window of 6,000 bp upstream and 80 bp downstream *RCAN3AS* exon 1 was analysed with different tools. Firstly, the Exon Finder software allowed us to identify a CpG island of 201 bp in length (GC, 81.5%) and a promoter region starting and ending, respectively, 622 bp and 52 bp upstream of the CpG island. Moreover, Neural Network Promoter Prediction server of BIOSINO showed a possible TSS in the first base downstream the identified CpG island, located 371 bp upstream the exon 1 start (according to BF448186 reference EST). This finding could suggest a 5′ elongation of *RCAN3AS* exon 1, considering also that the complementary strand in this portion results to be transcribed as demonstrated by BP326714 EST (referred to *RCAN3-1a,2,3,4,5* isoform, on the opposite strand).

### RCAN3AS protein isoforms

All four *RCAN3AS* isoforms have the same coding sequence (171 bp) on exon 3 (Figure 4B) that encodes for a predicted protein of 56 amino acids, with a theoretical pI/Mw of 4.70/6,356.42. This protein sequence has been deposited in GenBank by Venter et al. (2005), as a conceptual translation (#EAW95131).

By using BLASTP software, RCAN3AS sequence did not show similarity with any known protein or protein domain.

## Discussion

Human *RCAN3* [3] is mapped on chromosome 1 and belongs to the human *RCAN* gene family that includes *RCAN1*, mapping on chromosome 21 [1], and *RCAN2*, mapping on chromosome 6 [2]. All three genes can be considered multi-transcript systems, because of the amount of alternative isoforms described. The *RCAN1* gene consists of seven exons, amongst which exons 1-4 can be alternatively transcribed or spliced, thus producing different mRNA isoforms that encode for two major protein isoforms of 252 (RCAN1-1) [44] and 197 (RCAN1-4) amino acids [45], [46]. *RCAN2* consists of seven exons and produces three different transcripts (*RCAN2-1,3*, *RCAN2-2,3*, and *RCAN2-4*) and two related protein products (RCAN2-3 and RCAN2-4) [47]. *RCAN3* is a five-exon gene [20], [24] spanning 33,042 bp and encoding for a 241 amino acids protein (estimated molecular weight 27.5 kDa). Five *RCAN3* transcripts generated by alternative splicing have been identified to date (Figure 1) [3], [20], [22], as described in the Introduction section.

The denomination for *RCAN* genes (previously named *DSCR1L* genes) has been widely discussed in an international forum manuscript [4]. The name “Regulator of calcineurin” indicates that all three family members interact with and regulate calcineurin [5]-[7], a Ca^2+^/calmodulin-activated serine/threonine phosphatase that is involved in the transcriptional activation of many target genes. RCAN3 binding to calcineurin is exclusively mediated by a CIC motif, also present in RCAN1 and RCAN2. The CIC motif contains the ELHA calcineurin-binding sequence, which has been demonstrated to inhibit the NFAT-dependent cytokine gene expression in human T cell lines and, therefore, T cell activation [7], [8]. RCAN3-2,3,4,5, RCAN3-2,3,4b,5 and RCAN3-2,4,5 all contain the CIC motif, necessary for calcineurin binding. However, RCAN3-2,5 and RCAN3-2,3,5 isoforms do not include the functional CIC motif. It has been therefore hypothesized that they do not interact with calcineurin [7], [22]. With regard to the functional role of RCAN3, our group demonstrated that it interacts with TNNI3 [20], the inhibitory cardiac troponin preventing contraction in the absence of calcium and troponin C [21]. Exon 2 has been found to be sufficient for the binding of RCAN3 with TNNI3 [20]. Therefore, all alternative protein isoforms to date identified interact with TNNI3 [20], [22].  In the present work, a combination of *in silico* and *in vitro* analyses allowed us to explore the complexity of the human *RCAN3* locus, focusing on new human spliced isoforms, a new gene overlapping and in antisense with respect to *RCAN3*, and new *RCAN3* isoforms with different 5′UTR, whose expression is possibly regulated by alternative promoter sequences.

Here we describe 16 novel *RCAN3* mRNA isoforms. Four new isoforms were identified unexpectedly during *RCAN3-1,2,3,4,5* RT-PCR cloning: *RCAN3-1,3,4,5*, lacking exon 2, *RCAN3-1c,2,3,4,5*, lacking the last 33 bp of exon 1, *RCAN3-1c,3,4,5*, lacking exon 2 and the last 33 bp of exon 1 and *RCAN3-1c,2,4,5*, lacking exon 3 and the last 33 bp of exon 1 (Figure 1). Furthermore new isoforms of *RCAN3*, carrying other alternative first exons compared to the reference transcript *RCAN3-1,2,3,4,5*, were identified by sequence comparison with EST database entries and following search by Genome and EcGene Browsers. Two isoforms, containing alternative exon 1a, localized 434 bp upstream the reference exon 1, were cloned in human prostate tissue: a new long sequence *RCAN3-1a,2,3,4,5* and a spliced alternative isoform lacking the exon 2, named *RCAN3-1a,3,4,5* were found (Figure 1 and Figure 2A). The existence of the *RCAN3-1,3,4,5*, *RCAN3-1c,3,4,5* and *RCAN3-1a,3,4,5* isoforms were also confirmed by RT-PCR amplification using *RCAN3* specific forward primers and a common reverse primer. Moreover, a new isoform containing exon 1b, 4,443 bp downstream the reference exon 1, was identified in human lung and testis tissues (Figure 1 and Figure 2B). All four identified alternative first exons (1, 1c, 1a and 1b) are not coding and, in all isoforms containing the following exon 2 - exon 3 - exon 4 - exon 5 assembly, the coding sequence starts in exon 2, giving rise to the same protein product of 241 amino acids (RCAN3-2,3,4,5). The protein maintains both domains for TNNI3 binding and CIC motif to bind calcineurin (Figure 6). Instead, the three new isoforms *RCAN3-1,3,4,5*, *RCAN3-1c,3,4,5* and *RCAN3-1a,3,4,5* produce a protein product of 116 amino acids that lacks the exon 2 encoded domain, necessary for TNNI3 binding, while it maintains the CIC motif (Figure 6).

In order to demonstrate the possible presence of exon 1 in the previously identified isoforms *RCAN3-2,4,5*, *RCAN3-2,3,5*, *RCAN3-2,3,4b,5* and *RCAN3-2,5*, specific RT-PCR cloning experiments were performed revealing the existence of the isoforms *RCAN3-1,2,4*, *RCAN3-1,2,3,5* and *RCAN3-1,2,5*, and of the unexpected isoforms *RCAN3-1c,2,4*, *RCAN3-1c,2,3,5* and *RCAN3-1c,2,5* (Figure 1 and Figure 2C). Due to the specific isoform primer design, these findings do not allow us to assert with certainty that these alternative isoforms are referred to complete isoforms (extended up to exon 5), although this event is likely. On the other hand, the discovery of four alternative non-coding first exons prompted us to also perform RT-PCR experiments with specific forward and reverse primers, leading to the identification of *RCAN3-1a,2,4*, *RCAN3-1a,2,5*, *RCAN3-1a,2,3,5* and *RCAN3-1b,2,4* additional transcript assemblies (Figure 1). This confirmed the hypothesis that all the isoforms cloned and starting by exon 2 could be related to all the possible alternative first exons and not only to the previously known reference exon 1.

*RCAN3* locus complexity was enriched by the identification of *RCAN3AS* gene, located on the opposite strand compared to *RCAN3* and partially overlapping *RCAN3* exon 1a with its exon 1 (exon -1 in Figure 1). *RCAN3AS-1,2,3* isoform is composed by three exons and it has been cloned in human testis and pancreas tissues. Other three *RCAN3AS* isoforms (*RCAN3AS-1,2a,3, RCAN3-1,2b,3* and *RCAN3AS-1,3*) have been cloned in human pancreas. *RCAN3AS-1,2a,3* and *RCAN3AS-1,2b,3* consist of 3 exons, with an exon 2 5′ elongation of 52 bp and of 93 bp, respectively. *RCAN3AS-1,3* consists of 2 exons, since it lacks exon 2 completely (Figure 1 and Figure 4). The predicted protein encoded is the same for all *RCAN3AS* isoforms, since it is comprised in exon 3 and consists of 56 amino acids. Its sequence has no similarity with any human putative domain.

The comprehension of the genomic organization of *RCAN3AS* here presented represents the basis to carry out further functional studies in order to investigate its possible regulative role on *RCAN3*, also as a putative non-coding RNA (ncRNA).

Our findings bear out possibility that gene expression regulation in higher eukaryotes is enriched with the wide number of alternative spliced isoforms, with data on overlapping sequences (“overlapping genes”), sequences related to ncRNA and natural antisense RNA (NAT), whose presence at a gene locus identify a "multi-transcript” locus [48]. It has been estimated that 40-60% of all human genes and 74% of multiexon human genes are alternatively spliced [49]. These estimates do not take into account how many different alternatively spliced isoforms exist for any given gene [49]. Different mechanisms of alternative splicing could be identified in human genes, from lacking three bases from one exon, like in subtle splicing [50], [51], to lacking one or more discrete exons [52], like in our cases. All these mechanisms probably explain the functional complexity of vertebrates, as opposed to invertebrates [52].

To seek confirmation of new *RCAN3* and *RCAN3AS* isoforms evidence and to retrieve information about their expression, EST or defined cDNA (DNA complementary to RNA) sequences were searched in all organisms. In particular, in *Homo sapiens*, sequences referred to all alternative first exons - exon 2 junctions were retrieved, while no EST or defined cDNA sequences matching alternative first exons - exon 3 junctions were identified, possibly suggesting their low expression. Many ESTs containing exon 1 or 1c - exon 2 sequences were assigned to *RCAN3-1,2,3,4,5* and *RCAN3-1c,2,3,4,5* cDNAs, respectively (Table 2). With the exception of *RCAN3-1,2,4,5* and *RCAN3-1c,2,4,5*, no human ESTs referred to previously identified alternative *RCAN3* isoforms [3], [20], [22] (Figure 1), were retrieved. The low expression level of previously described alternative isoforms [22] and the absence of *RCAN3* ESTs containing alternative first exons (1, 1c, 1a or 1b) could explain the difficulty to clone their complete 5′ sequences. Human sequences referred to *RCAN3AS* isoforms were retrieved, except for *RCAN3AS-1,3* (Table 3). Identical results for *RCAN3* and *RCAN3AS* genes were obtained using ECgene Browser and Genome Browser analyses.

Analyses of new *RCAN3* isoforms mRNA allowed us to identify three previously described *RCAN3* SNPs [22], already registered in the single nucleotide polymorphism database. SNPs, now numbering almost five million entries for the human genome, are an increasingly important tool for studying the structure and history of the human genome as well as human diseases [53]. For all new *RCAN3* isoforms the presence of the three SNPs, responsible of a G→A change, does not modify their predicted amino acid sequences (Table S1).

On the basis of our previous observation that the *RCAN3* gene appears to be expressed in several tissue types [3], a qualitative systematic analysis by RT-PCR was performed. We demonstrated the presence of *RCAN3-1a,2,3,4,5*, *RCAN3-1b,2,3,4,5* and *RCAN3AS-1,2,3* isoforms in 17 normal human tissues investigated, and their particular expression in tissues used for cloning experiments, according to reference EST sources. A qualitative comparison between *RCAN3-1a,2,3,4,5* and *RCAN3AS-1,2,3* RT-PCR expression panels, obtained at the maximum distance from the PCR reaction plateau, allow us to hypothesize a possible relative regulation. Similar experiments performed to verify expression of all isoforms containing exon 1 - exon 2 or exon 1c - exon 2 junctions revealed a similar expression pattern (Figure 3A and B), thus indicating a stochastic use of exon 1 or exon 1c, as well as in some known subtle splicing mechanisms. In fact, stochastic splice site selection during developmental stages or in tissues and constant splicing ratios indicate that different functions are not always associated with differential splicing [38].

Particular attention was given to the new evidence of four *RCAN3* alternative non-coding first exons (exon 1, 1c, 1a and 1b), a phenomenon that adds importance to the complexity of this mammalian gene structure. Alternative non-coding regulative regions in 5′UTR could be linked to the use of alternative gene promoters, resulting in tissue-specific or developmental stage-specific gene expression regulation [54]. In fact, a recent annotation suggested that almost 50% of the protein-coding genes contain alternative promoters [55]. Therefore, an analysis of human *RCAN3* genomic sequence was performed to investigate the possible presence of alternative promoters referred to the alternative first exons (Figure 5A). Only one CpG island of 202 bp in length was found by First Exon Finder software upstream and near the exon 1 start of the *RCAN3* gene. The identification by Promoter Prediction software of three hypothetical short sequences containing TSSs within the CpG island could allow us to hypothesize its role as housekeeping gene. In fact, all housekeeping and widely expressed genes have a CpG island covering the transcription start, whereas only 40% of the genes with a tissue-specific or limited expression are associated with islands [56], [57]. Although no transcript with first exon 1 or 1c has been obtained with RACE method, one of the four short sequence containing a possible TSS (2,964–2,966 nt positions of AL034582) is very close to the first nucleotide of the reference sequence (NM_013441) referred to the *RCAN3-1,2,3,4,5* isoform. NM_013441 sequence assembly has been conducted considering the complete 5′ *RCAN3* sequence derived from a massive sequencing project whose purpose was to clone full human ORFs from libraries enriched for full-length cDNAs [24]. Therefore, we could speculate that the predicted sequence containing the described TSS corresponds to a possible real transcription start region.

First Exon Finder software detected a promoter region upstream of exon 1a and ending within the same exon, whose possible TSS has been verified by RACE method. To date, the many tests performed with the RACE method allowed us to obtain only the possible TSS of the *RCAN3-1a,2,3,4,5* isoform. The retrievement of this isoform has been favored by the selected tissue (prostate) where *“RCAN3-1a*” isoforms are particularly expressed. However, further investigations will be needed in order to study *RCAN3* alternative promoters.

Manual analysis of the genomic sequence upstream of exon 1a revealed a possible guanine repetition responsible of a G-quadruplex structure formation [40]. G-quadruplexes are four-stranded DNA structure formed from repetitions of three or four adjacent guanines (G-tracts) in presence of monovalent cations such as K^+^ and Na^+^. Both prokaryotic and eukaryotic genomes from yeast to man are rich in G-quadruplexes. The highest occurrence of G-quadruplexes is present in repetitive DNA regions such as telomeres and, interestingly, up to 40% of human gene promoters [41]. Furthermore, RNAs are known to form non-canonical structures such as triple-strands and G-quadruplexes located in their 5′UTRs. The regulated formation of these structures in the promoter region has been demonstrated to provide an elegant nucleic-acid-based mechanism for modulating transcription and translation [41]. A promoter region was not retrieved upstream of exon 1b by Promoter Prediction software, but manual analysis underlines the presence of a TATA box and of a CAAT box at a coherent distance. On the other hand, the Promoter Prediction software showed a TSS site in a position plausible with the 5′UTR average length of human genes [42]. A similar analysis of genomic sequences referred to *RCAN3AS* isoforms allowed us to identify a possible common promoter region, a CpG island and a plausible TSS site, upstream of *RCAN3AS* exon 1 (Figure 5B). Moreover, the presence of four alternative *RCAN3AS* 5′UTR and of the shared downstream coding sequence indicates a possible 5′UTR different use, due to a specific regulative role.

The interest in performing a comparative genomic analysis of alternative first exons led us to search them in all organisms. Only sequences similar to human exon 1 - exon 2 (defined cDNAs, but not EST sequences), human exon 1c - exon 2 and exon 1c - exon 3 (only 1 EST) sequences were identified. No sequences referred to human exon 1 - exon 3, exon 1a - exon 2 or 3 and exon 1b - exon 2 sequences were retrieved, but the possibility of their assembly in the transcription process could be hypothesized after manual genomic sequence analyses of some primates and some mammals-not primates (Table S2). A similar analysis was performed for all human *RCAN3AS* isoforms and any relative sequence was retrieved in other organisms.

The study in *Homo sapiens* of splice donor sequences for all possible alternative first exons - first intron - exon 2 or 3 junctions showed, in all cases, a high sequence conservation compared to the consensus reference sequence. In particular, the splice donor sequence is more adherent when isoforms containing exon 1c are transcribed, compared to isoforms containing exon 1 (Figure S1). A similar study was performed to analyze *RCAN3AS* exon 1 - first intron - alternative exon 2 or exon 3 genomic sequences. In all sequences, the splice acceptor sequences were very conserved (Figure S3).

In order to retrieve sequences related to human *RCAN3-1,2,3,4,5* and *RCAN3-1c,2,3,4,5* isoforms, a detailed bioinformatic searches in nr and EST databases were performed in all organisms (Table S3). With regard to *RCAN3-1,2,3,4,5*, transcript models were only found in primates. On the contrary, transcript models and transcribed sequences referred to *RCAN3-1c,2,3,4,5* were found in primates, mammals - not primates and not mammals (Table S3, second column). The study of the donor splicing sequences on all investigated genomic sequences (Table S3, third column) showed the presence of high sequence conservation compared to consensus, especially in the exon 1c - exon 2 formation (Figure S2). Moreover, in primates both donor signal sites were visible on genomic sequences, thus suggesting their alternative possible use, like it happens in *Homo sapiens*. In mammals-not primates, only the donor splice site corresponding to human exon 1c was retrieved, with consensus sequence identity of 100%. In these organisms a splice donor site related to human exon 1 was not present. Even in organisms not belonging to the class of mammals (*Gallus gallus* and *Danio rerio*), analysis of genomic sequences indicated that only a splice site similar to human exon 1c, whose signal sequences are highly conserved, would be used. These genomic contexts, associated with the failure to actually find transcribed sequences for exon 1 in mammals-not primates and in not mammals, suggest that in these species there is not the possibility of alternative use of exon 1 or 1c, but only of an exon equivalent to human 1c. Therefore, from an evolutionary point of view, the splice site exon 1c-like appears to be older, as it is the only one conserved in all species analyzed and characterized by splice donor sequences that often perfectly match the consensus sequence, thus indicating a strong splicing signal.

In the present work we have demonstrated the existence of a complex *RCAN3* multi-transcript locus, which consists of 21 alternative *RCAN3* isoforms and of 4 isoforms of a new identified antisense and overlapping gene (*RCAN3AS*). Analyses of all transcripts and their putative proteins, of their expression patterns and of their regulatory non-coding regions, are important to clarify the genomic structure and the evolutionary pathway of the *RCAN3* gene, as well as giving an important basis for further functional experiments. We think that the discovery and the analysis of alternative first non-coding exons is of primary interest to study the regulative role of *RCAN3* 5′UTR in different tissues and/or specific physiological and pathological cellular conditions. Finally, the complexity of the *RCAN3* locus has been enriched by the newly identified *RCAN3AS* gene, whose study could be useful for the comprehension of *RCAN3* gene regulation, as well as for the new human gene role itself.

## Supporting Information

Figure S1 **Splice consensus sequences comparison (alternative** ***RCAN3*** **first exons) in** ***Homo sapiens*** **.** The analysis was carried out for different *RCAN3* isoforms aligning alternative exons 1 - first intron - exon 2 or 3 (according to considered isoform). CAGGTRAGT: splice donor consensus sequence; R =  purine A/G. YAGN: splice acceptor consensus sequence; Y =  pyrimidine C/T; N =  all nucleotides. In bold black: splicing donor nucleotides identity with consensus sequence and underlined GT alternative splice signals. In bold white: splicing acceptor nucleotides identity with consensus sequence. In bold grey: splicing donor or acceptor nucleotides without identity with consensus sequence. In bold grey and underlined: purine/purine or pyrimidine/pyrimidine base pair substitution compared to consensus sequence.
(XLS)

Figure S2 **Splice consensus sequences comparison (alternative** ***RCAN3*** **exon 1 and 1c) in different species.** * Alternative first exons and exon 2 of studied species were assembled for similarity with sequences of related species (*Homo sapiens* transcript used for *Macaca mulatta*, *Macaca mulatta* transcript used for *Callithrix jacchus*, *Canis lupus familiaris* transcript used for *Ailuropoda melanoleuca* and *Mus musculus* transcript used for *Rattus norvegicus*). CAGGTRAGT: splice donor consensus sequence; R =  purine A/G. YAGN: splice acceptor consensus sequence; Y =  pyrimidine C/T; N =  all nucleotides. Dark grey highlighted: conserved donor and acceptor splice sequences. In bold black: splicing donor nucleotides identity with consensus sequence. In bold white: splicing acceptor nucleotides identity with consensus sequence and other AG alternative splice signals. In bold grey: splicing donor or acceptor nucleotides without identity with consensus sequence. In bold black underlined: ATG start codon. White block: Primates; light grey block: Mammals, not Primates; medium grey block: not Mammals.
(XLS)

Figure S3 **Splice consensus sequences comparison between** ***RCAN3AS*** **isoforms in** ***Homo sapiens.*** *RCAN3AS* gene isoforms are on opposite strand compared to *RCAN3* gene. Here relative genomic sequences are reported in 5′-3′ direction. CAGGTRAGT: splice donor consensus sequence; R =  purine A/G. YAGN: splice acceptor consensus sequence; Y =  pyrimidine C/T; N =  all nucleotides. In bold black: splicing donor nucleotides identity with consensus sequence. In bold white: splicing acceptor nucleotides identity with consensus sequence. Double underlined: exon 2 splice acceptor sequence. Single underlined: exon 2a splice acceptor sequence. In bold grey: splicing donor or acceptor nucleotides without identity with consensus sequence.
(XLS)

Table S1 **Single nucleotide polymorphisms (SNPs) of new** ***RCAN3*** **isoforms.** ^a^ Base pair in brackets referred to the corresponding GenBank sequence (“GenBank accession no.” column).
(PDF)

Table S2 **Alternative first exons in other species.** Search was performed in all organisms with BLASTN software (default parameters, with no filter, excluding *Homo sapiens*). For all human *RCAN3* isoforms a specific alternative first exon - exon 2 or a specific alternative first exon - exon 3 query sequences were used. Manual analysis of found sequences allowed us to assign them to a specific isoform. “nr” (non redundant), “nt” (nucleotide), “refseq_rna” (reference sequence) and “EST” (expressed sequence tag).
(PDF)

Table S3 ***RCAN3-1,2,3,4,5*** **and** ***RCAN3-1c,2,3,4,5*** **detailed comparison in other species.** Search was performed in all organisms with BLASTN and TBLASTN software (default parameters, with no filter). Human NM_013441 and NP_038469 were used as initial query sequence to retrieve other organism transcripts. “*” indicates transcripts attributed to *RCAN3-1,2,3,4,5* or *RCAN3-1c,2,3,4,5* isoforms after a comparison between genomic sequence of studied species and reference transcript sequence of a species evolutionarily related. For different species more complete retrieved transcript sequence were used as reference to query refseq_genomic or other genomic sequences databases at NCBI. Specie-specific exon 1-exon 2 or exon 1c-exon 2 assembly were used as query to search by BLASTN the specific organism ESTs in order to prove the expression of the relative isoforms and to complete the 5′ transcript ends. “nr” (non redundant), “nt” (nucleotide), “EST” (human expressed sequence tag).
(PDF)

## References

[1] Fuentes JJ, Pritchard MA, Planas AM, Bosch A, Ferrer I (1995). A new human gene from the Down syndrome critical region encodes a proline-rich protein highly expressed in fetal brain and heart.. Hum Mol Genet.

[2] Miyazaki T, Kanou Y, Murata Y, Ohmori S, Niwa T (1996). Molecular cloning of a novel thyroid hormone-responsive gene, ZAKI-4, in human skin fibroblasts.. J Biol Chem.

[3] Strippoli P, Lenzi L, Petrini M, Carinci P, Zannotti M (2000). A new gene family including DSCR1 (Down Syndrome Candidate Region 1) and ZAKI-4: characterization from yeast to human and identification of DSCR1-like 2, a novel human member (DSCR1L2).. Genomics.

[4] Davies KJ, Ermak G, Rothermel BA, Pritchard M, Heitman J (2007). Renaming the DSCR1/Adapt78 gene family as RCAN: regulators of calcineurin.. FASEB J.

[5] Fuentes JJ, Genesca L, Kingsbury TJ, Cunningham KW, Perez-Riba M (2000). DSCR1, overexpressed in Down syndrome, is an inhibitor of calcineurin-mediated signaling pathways.. Hum Mol Genet.

[6] Rothermel B, Vega RB, Yang J, Wu H, Bassel-Duby R (2000). A protein encoded within the Down syndrome critical region is enriched in striated muscles and inhibits calcineurin signaling.. J Biol Chem.

[7] Mulero MC, Aubareda A, Schluter A, Perez-Riba M (2007). RCAN3, a novel calcineurin inhibitor that down-regulates NFAT-dependent cytokine gene expression.. Biochim Biophys Acta.

[8] Aubareda A, Mulero MC, Perez-Riba M (2006). Functional characterization of the calcipressin 1 motif that suppresses calcineurin-mediated NFAT-dependent cytokine gene expression in human T cells.. Cell Signal.

[9] Bueno OF, Lips DJ, Kaiser RA, Wilkins BJ, Dai YS (2004). Calcineurin Abeta gene targeting predisposes the myocardium to acute ischemia-induced apoptosis and dysfunction.. Circ Res.

[10] Molkentin JD, Lu JR, Antos CL, Markham B, Richardson J (1998). A calcineurin- dependent transcriptional pathway for cardiac hypertrophy.. Cell.

[11] Aramburu J, Rao A, Klee CB (2000). Calcineurin: from structure to function.. Curr Top Cell Regul.

[12] Winslow MM, Neilson JR, Crabtree GR (2003). Calcium signalling in lymphocytes.. Curr Opin Immunol.

[13] Chin ER, Olson EN, Richardson JA, Yang Q, Humphries C (1998). A calcineurin- dependent transcriptional pathway controls skeletal muscle fiber type.. Genes Dev.

[14] Parsons SA, Millay DP, Wilkins BJ, Bueno OF, Tsika GL (2004). Genetic loss of calcineurin blocks mechanical overload-induced skeletal muscle fiber type switching but not hypertrophy.. J Biol Chem.

[15] Zeng H, Chattarji S, Barbarosie M, Rondi-Reig L, Philpot BD (2001). Forebrain-specific calcineurin knockout selectively impairs bidirectional synaptic plasticity and working/episodic- like memory.. Cell.

[16] Lee JI, Ahnn J (2004). Calcineurin in animal behavior.. Mol Cells.

[17] Kim YS, Lee HJ, Jang C, Kim HS, Cho YJ (2009). Knockdown of RCAN1.4 Increases Susceptibility to FAS-mediated and DNA-damage-induced Apoptosis by Upregulation of p53 Expression.. Korean J Physiol Pharmacol.

[18] Sales KJ, Grant V, Cook IH, Maldonado-Perez D, Anderson RA (2010). Interleukin-11 in endometrial adenocarcinoma is regulated by prostaglandin F2alpha-F-prostanoid receptor interaction via the calcium-calcineurin-nuclear factor of activated T cells pathway and negatively regulated by the regulator of calcineurin-1.. Am J Pathol.

[19] Canaider S, Vettraino M, Norling LV, Spisni E, Facchin F (2010). Human RCAN3 gene expression and cell growth in endothelial cells.. Int J Mol Med.

[20] Canaider S, Facchin F, Griffoni C, Casadei R, Vitale L (2006). Proteins encoded by human Down syndrome critical region gene 1-like 2 (DSCR1L2) mRNA and by a novel DSCR1L2 mRNA isoform interact with cardiac troponin I (TNNI3).. Gene.

[21] Cummins P, Perry SV (1978). Troponin I from human skeletal and cardiac muscles.. Biochem J.

[22] Facchin F, Canaider S, Vitale L, Frabetti F, Griffoni C (2008). Identification and analysis of human RCAN3 (DSCR1L2) mRNA and protein isoforms.. Gene.

[23] Strippoli P, D'Addabbo P, Lenzi L, Giannone S, Canaider S (2002). Segmental paralogy in the human genome: a large-scale triplication on 1p, 6p, and 21q.. Mamm Genome.

[24] Strausberg RL, Feingold EA, Grouse LH, Derge JG, Klausner RD (2002). Generation and initial analysis of more than 15,000 full-length human and mouse cDNA sequences.. Proc Natl Acad Sci USA.

[25] Engels WR (1993). Contributing software to the internet: the Amplify program.. Trends Biochem Sci.

[26] Sharrocks AD, Griffin HG, Griffin AM (1994). The design of primer for PCR.. PCR Technology - Current Innovations.

[27] Davis LG, Kuehl WM, Battey JF (1994). Basic Methods in Molecular Biology..

[28] NCBI (National Center for Biotechnology Information) homepage Available: http://www.ncbi.nlm.nih.gov/, last accessed on 15/09/2010

[29] Kim N, Shin S, Lee S (2005). ECgene: genome-based EST clustering and gene modeling for alternative splicing.. Genome Res.

[30] Hinrichs AS, Karolchik D, Baertsch R, Barber GP, Bejerano G (2006). The UCSC Genome Browser Database: update 2006.. Nucleic Acids Res.

[31] ClustalW homepage Available: http://www.ebi.ac.uk/clustalw, last accessed on 10/10/2010

[32] Hiller M, Nikolajewa S, Huse K, Szafranski K, Rosenstiel P (2007). TassDB: a database of alternative tandem splice sites.. Nucleic Acids Res.

[33] Neural Network Promoter Prediction version 2.2 at Berkeley Drosophila Genome Project homepage Available: http://www.fruitfly.org/seq_tools/promoter.html, last accessed on 10/10/2010

[34] Neural Network Promoter Prediction Server at BIOSINO (Bioinformation Center of Shanghai Institutes for Biological Science Chinese Academy of Sciences) homepage Avaiable: http://Promoter.biosino.org, last accessed on 10/10/2010

[35] Davuluri RV, Grosse I, Zhang MQ (2001). Computational identification of promoters and first exons in the human genome.. Nat Genet.

[36] GenBank at NCBI homepage Available: http://www.ncbi.nlm.nih.gov/Genbank, last accessed on 15/09/2010

[37] Mader RM, Schmidt WM, Sedivy R, Rizovski B, Braun J (2001). Reverse transcriptase template switching during reverse transcriptase-polymerase chain reaction: artificial generation of deletions in ribonucleotide reductase mRNA.. J Lab Clin Med.

[38] Hiller M, Platzer M (2008). Widespread and subtle: alternative splicing at short-distance tandem sites.. Trends Genet.

[39] Single Nucleotide Polymorphism Database homepage Available: http://www.ncbi.nlm.nih.gov/entrez/query.fcgi?db=snp, last accessed on 15/09/2010

[40] Lipps HJ, Rhodes D (2009). G-quadruplex structures: in vivo evidence and function.. Trends Cell Biol.

[41] Huppert JL, Balasubramanian S (2007). G-quadruplexes in promoters throughout the human genome.. Nucleic Acids Res.

[42] Chatterjee S, Pal JK (2009). Role of 5′- and 3′-untranslated regions of mRNAs in human diseases.. Biol Cell.

[43] Strippoli P, Fantoni A, Bozzaro S, Del Sal G, Ferrari S, Tripodi M (2009). Analisi del genoma umano.. Biologia cellulare e genetica.

[44] Casadei R, Strippoli P, D'Addabbo P, Canaider S, Lenzi L (2003). mRNA 5′ region sequence incompleteness: a potential source of systematic errors in translation initiation codon assignment in human mRNAs.. Gene.

[45] Fuentes JJ, Pritchard MA, Estivill X (1997). Genomic organization, alternative splicing, and expression patterns of the DSCR1 (Down syndrome candidate region 1) gene.. Genomics.

[46] Genesca L, Aubareda A, Fuentes JJ, Estivill X, De La Luna S (2003). Phosphorylation of calcipressin 1 increases its ability to inhibit calcineurin and decreases calcipressin half-life.. Biochem J.

[47] Cao X, Kambe F, Miyazaki T, Sarkar D, Ohmori S (2002). Novel human ZAKI-4 I soforms: hormonal and tissue-specific regulation and function as calcineurin inhibitors.. Biochem J.

[48] Carninci P, Yasuda J, Hayashizaki Y (2008). Multifaceted mammalian transcriptome.. Curr Opin Cell Biol.

[49] Kim H, Klein R, Majewski J, Ott J (2004). Estimating rates of alternative splicing in mammals and invertebrates.. Nat Genet.

[50] Vitale L, Lenzi L, Huntsman SA, Canaider S, Frabetti F (2006). Differential expression of alternatively spliced mRNA forms of the insulin-like growth factor 1 receptor in human neuroendocrine tumors.. Oncol Rep.

[51] Vitale L, Frabetti F, Huntsman SA, Canaider S, Casadei R (2007). Sequence, "subtle" alternative splicing and expression of the CYYR1 (cysteine/tyrosine-rich 1) mRNA in human neuroendocrine tumors.. BMC Cancer.

[52] Blencowe BJ (2006). Alternative splicing: new insights from global analyses.. Cell.

[53] Riva A, Kohane IS (2004). A SNP-centric database for the investigation of the human genome.. BMC Bioinformatics.

[54] Baek D, Davis C, Ewing B, Gordon D, Green P (2007). Characterization and predictive discovery of evolutionarily conserved mammalian alternative promoters.. Genome Res.

[55] Davuluri RV, Suzuki Y, Sugano S, Plass C, Huang TH (2008). The functional consequences of alternative promoter use in mammalian genomes.. Trends Genet.

[56] Larsen F, Gundersen G, Lopez R, Prydz H (1992). CpG islands as gene markers in the human genome.. Genomics.

[57] Zhu J, He F, Hu S, Yu J (2008). On the nature of human housekeeping genes.. Trends Genet.

