# Supplementary material for: Complexity of Bidirectional Transcription and Alternative Splicing at Human RCAN3 Locus
Source: PLoS One. 2011 Sep 22;6(9):e24508. doi: 10.1371/journal.pone.0024508 (PMC3178534; doi:10.1371/journal.pone.0024508)
Supplement: Table S1 — Single nucleotide polymorphisms (SNPs) of new RCAN3 isoforms. a Base pair in brackets referred to the corresponding GenBank sequence (“GenBank accession no.” column). (PDF) [file pone.0024508.s004.pdf]

Table S1. Single nucleotide polymorphisms (SNPs) of new *RCAN3* isoforms

***RCAN3* isoforms**	**GenBank accession no.**	**dbSNP cluster: rs196429**	**dbSNP cluster: rs196430**	**dbSNP cluster: rs196432**
RCAN3-1,2,3,4,5	NM_013441	G (727 bp) [a]	G (775 bp)	G (976 bp)
RCAN3-1,2,3,4,5	BC035854	A (727 bp)	A (775 bp)	A (976 bp)
RCAN3-1,3,4,5	HQ287726	G (256 bp)	G (304 bp)	G (505 bp)
RCAN3-1c,2,3,4,5	HQ317426	G (477 bp)	G (525 bp)	A (726 bp)
RCAN3-1c,3,4,5	HQ317427	G (223 bp)	G (271 bp)	G (472 bp)
RCAN3-1a,2,3,4,5	HQ317421	A (562 bp)	A (610 bp)	G (811 bp)
RCAN3-1a,3,4,5	HQ317422	A (308 bp)	G (356 bp)	A (557 bp)
RCAN3-1b,2,3,4,5	HQ317423	A (608 bp)	A (656 bp)	A (857 bp)

[a] Base pair in brackets referred to the corresponding GenBank sequence ("GenBank accession no." column)
