# Supplementary material for: Complexity of Bidirectional Transcription and Alternative Splicing at Human RCAN3 Locus
Source: PLoS One. 2011 Sep 22;6(9):e24508. doi: 10.1371/journal.pone.0024508 (PMC3178534; doi:10.1371/journal.pone.0024508)
Supplement: Table S2 — Alternative first exons in other species. Search was performed in all organisms with BLASTN software (default parameters, with no filter, excluding Homo sapiens). For all human RCAN3 isoforms a specific alternative first exon - exon 2 or a specific alternative first exon - exon 3 query sequences were used. Manual analysis of found sequences allowed us to assign them to a specific isoform. “nr” (non redundant), “nt” (nucleotide), “refseq_rna” (reference sequence) and “EST” (expressed sequence tag). (PDF) [file pone.0024508.s005.pdf]

Table S2. Alternative first exons in other species

cDNA	nr/nt or refseq_rna others	EST others	genomic sequence others
RCAN3-1,2,3,4,5	*Pan troglodytes*: XM_001167542	None	Listed in Table S3
	Pongo abelii: XM_002811276		
RCAN3-1,3,4,5	None	None	Listed in Table S3
RCAN3-1a,2,3,4,5	None	None	*Pongo abelii*: NW_002875515
			Callithrix jacchus: NW_003183707
RCAN3-1a,3,4,5	None	None	*Pongo abelii*: NW_002875515
			Callithrix jacchus: NW_003183707
RCAN3-1b,2,3,4,5	None	None	*Pan troglodytes*: NW_001230294, AADA01267496
			Pongo abelii: NW_002875515
			Macaca mulatta: NW_001111036, AANU01188091
			Callithrix jacchus: NW_003183707, ACFV01133095
			Loxodonta africana: AAGU03057323

			Ailuropoda melanoleuca: ACTA01113708
			Equus caballus: AAWR02028373
			Bos taurus: AC156403, DAAA02006408
			Pteropus vampyrus: ABRP01237998
			Canis lupus familiaris: CE645708, AAEX02023698
RCAN3-1c,2,3,4,5	Pan troglodytes: XM_001167438, XM_513206, XM_001167462		Listed in Table S3
	Pongo abelii: XM_002811275		
		Papio anubis: EY277657	
	Bos taurus: NM_001045945	Bos taurus: EH159774, EE890946, CB418553, DV800730, DY169856, DV840262, DY196129	
	Sus scrofa: AK345639, AK349452	Sus scrofa: EW106490, CJ017081, BP141896, FS697917, FS658919, FS713196, FS705492, FS705395, FS700296, DB789326, BW977154, BP440110, BP167063, FS698073, FS701530, DB805081, FS707785, FS690114, FS712957, FS712198,	

		BW974719, FS705422, BW968935, CJ002637, FS692423, FS709612, FS706765, BW977041, FS709837, CJ007783, FS674466, CJ036933, BP455127, BP171584, FS713336, EW387620, FS718176	
	Canis lupus familiaris: XM_544495	*Canis lupus familiaris*: DN375624	
RCAN3-1c,3,4,5	None	*Sus scrofa*: BW970272	Listed in Table S3
