# Supplementary material for: Complexity of Bidirectional Transcription and Alternative Splicing at Human RCAN3 Locus
Source: PLoS One. 2011 Sep 22;6(9):e24508. doi: 10.1371/journal.pone.0024508 (PMC3178534; doi:10.1371/journal.pone.0024508)
Supplement: Table S3 — RCAN3-1,2,3,4,5 and RCAN3-1c,2,3,4,5 detailed comparison in other species. Search was performed in all organisms with BLASTN and TBLASTN software (default parameters, with no filter). Human NM_013441 and NP_038469 were used as initial query sequence to retrieve other organism transcripts. “*” indicates transcripts attributed to RCAN3-1,2,3,4,5 or RCAN3-1c,2,3,4,5 isoforms after a comparison between genomic sequence of studied species and reference transcript sequence of a species evolutionarily related. For different species more complete retrieved transcript sequence were used as reference to query refseq_genomic or other genomic sequences databases at NCBI. Specie-specific exon 1-exon 2 or exon 1c-exon 2 assembly were used as query to search by BLASTN the specific organism ESTs in order to prove the expression of the relative isoforms and to complete the 5′ transcript ends. “nr” (non redundant), “nt” (nucleotide), “EST” (human expressed sequence tag). (PDF) [file pone.0024508.s006.pdf]

Table S3. ***RCAN3-1,2,3,4,5*** **and** ***RCAN3-1c,2,3,4,5*** **detailed comparison in other species**

cDNA	Reference nr/nt others	Relative genomic reference sequence	Relative ESTs
RCAN3-1,2,3,4,5	*Pan troglodytes*: XM_001167542	*Pan troglodytes*: NW_001230294	None
	Pongo abelii: XM_002811276	*Pongo abelii*: NW_002875515	
	*Macaca mulatta**: XM_001113362	*Macaca mulatta*: NW_001111099 NW_001111036	
	*Callithrix jacchus**: XM_002750444	*Callithrix jacchus*: NW_003183707	
RCAN3-1c,2,3,4,5	*Pan troglodytes*: XM_001167438	*Pan troglodyites*: NW_001230294	
	Pongo abelii: XM_002811275	*Pongo abelii*: NW_002875515	
	*Macaca mulatta**: XM_001113362	*Macaca mulatta*: NW_001111099, NW_001111036	
	*Callithrix jacchus**: XM_002750444	*Callithrix jacchus*: NW_003183707	
	*Ailuropoda melanoleuca**: XM_002913282	*Ailuropoda melanoleuca:* NW_003217307	
	Bos taurus: NM_001045945	*Bos taurus*: NW_003103851	*Bos taurus*: EH159774, EE890946, CB418553, DV840262, DY196129, DV800730, DY169856
	Sus scrofa: AK345639	*Sus scrofa*: CU972460	*Sus scrofa*: EW106490, CJ017081, BP141896, FS697917, FS658919, FS713196, FS705492, FS705395, FS700296, DB789326, BW977154, BP440110, BP167063, FS698073, FS701530, DB805081, FS707785, FS690114, FS712957, FS712198, BW974719, FS705422, BW968935, CJ002637, FS692423, FS709612, FS706765, BW977041, FS709837,

			CJ007783, FS674466, CJ036933, BP455127, BP171584, FS713336, EW387620, FS718176, BP439743, EB421270, FS661279, FS663793, CV866456
	Canis lupus familiaris: XM_544495	*Canis lupus familiaris*: NW_876292, NC_006584	*Canis lupus familiaris*: DN375624
	Mus musculus: NM_022980	*Mus musculus*: NT_039267	*Mus musculus*: BB641796, AA414395, BB639047, BY192142, BF463754, BE994224, BE984311, AI854381, AI842260, AI666466, BY246936, BF021307
	*Rattus norvegicus**: NM_001012746	*Rattus norvegicus*: NW_001084844	
	Gallus gallus: NM_001030907	*Gallus gallus*: NW_001471589	*Gallus gallus*: CO772075, AJ447031, AM068641, BM491687, BU245031, CN224613, AJ738137, BU294280, BU205026, CO503258
	Danio rerio: NM_001005392	*Danio rerio*: NW_001877781	*Danio rerio*: CF996050
